# Validation of Normalizations, Scaling, and Photofading Corrections for FRAP Data Analysis

**DOI:** 10.1371/journal.pone.0127966

**Published:** 2015-05-27

**Authors:** Minchul Kang, Manuel Andreani, Anne K. Kenworthy

**Affiliations:** 1 School of Science, Technology & Engineering Management, St. Thomas University, Miami Gardens, Florida, USA; 2 Department of Molecular Physiology and Biophysics, Vanderbilt University School of Medicine, Nashville, Tennessee, USA; German Cancer Research Center, GERMANY

## Abstract

Fluorescence Recovery After Photobleaching (FRAP) has been a versatile tool to study transport and reaction kinetics in live cells. Since the fluorescence data generated by fluorescence microscopy are in a relative scale, a wide variety of scalings and normalizations are used in quantitative FRAP analysis. Scaling and normalization are often required to account for inherent properties of diffusing biomolecules of interest or photochemical properties of the fluorescent tag such as mobile fraction or photofading during image acquisition. In some cases, scaling and normalization are also used for computational simplicity. However, to our best knowledge, the validity of those various forms of scaling and normalization has not been studied in a rigorous manner. In this study, we investigate the validity of various scalings and normalizations that have appeared in the literature to calculate mobile fractions and correct for photofading and assess their consistency with FRAP equations. As a test case, we consider linear or affine scaling of normal or anomalous diffusion FRAP equations in combination with scaling for immobile fractions. We also consider exponential scaling of either FRAP equations or FRAP data to correct for photofading. Using a combination of theoretical and experimental approaches, we show that compatible scaling schemes should be applied in the correct sequential order; otherwise, erroneous results may be obtained. We propose a hierarchical workflow to carry out FRAP data analysis and discuss the broader implications of our findings for FRAP data analysis using a variety of kinetic models.

## Introduction

**Fig 1:**
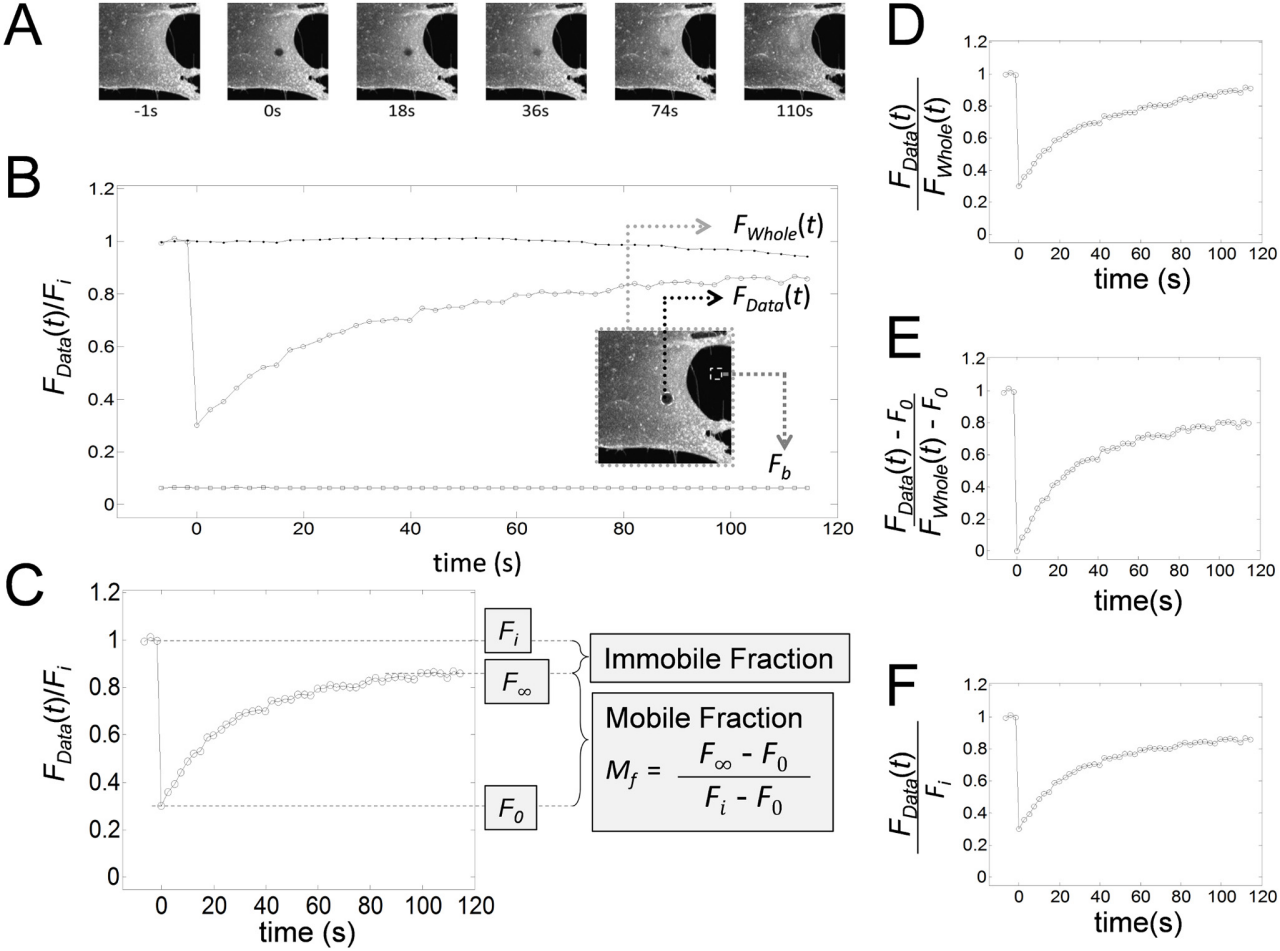
Representative images and data from a confocal FRAP experiment, and examples of commonly used normalizations and scalings applied to FRAP data. (A) Representative images from a FRAP experiment on Alexa488-CTxB. (B) Mean fluorescence intensity (*N* = 13) from the bleaching ROI (∘, *F*_*Data*_(*t*)), whole image (•, *F*_*Whole*_(*t*)), and background (▫) from a FRAP experiment of Alexa488-CTxB. The image in the inset shows the locations where *F*_*Data*_(*t*) (∘) and background (▫) were measured. (C) In FRAP analysis, prebleach steady state, postbleach initial, and postbleach steady state fluorescence intensities are typically denoted as *F*_*i*_, *F*_0_, and *F*_∞_. These parameters can be used to calculate the mobile fraction (*M*_*f*_) and immobile fraction (1−*M*_*f*_) from the normalized FRAP data (*F*_*Data*_(*t*)/*F*_*i*_) as indicated in the boxed equation. (D)–(F) The same FRAP curve was subjected to different scaling schemes, including an exponential scaling (D), an affine scaling (E), and a linear scaling (F).

Over the past few decades, Fluorescence Recovery After Photobleaching (FRAP) has become an indispensable biophysical tool for tracking cellular organelles, proteins, and lipids in cells in a spatio-temporal manner [1–7]. Over the course of those years, there have been considerable advances in microscope technology. However, the basic principle of FRAP remains the same. In diffusion FRAP, fluorescently tagged molecules in a small region of interest (ROI) are irreversibly photobleached using a high intensity laser source for a short period of time, and then the exchange of fluorescent and photobleached molecules in and out of the bleached region is monitored using low intensity laser excitation to follow fluorescence recovery. In this process, the microscope system records the fluorescence intensity in a relative scale (for example 8 bit images: 0 ∼ 256 scale) and generates a series of fluorescence images (Fig 1A). The fluorescence intensity in the bleached ROI is then collected and plotted as a function of time to produce a FRAP recovery curve (Fig 1B). In this curve, *F*_*i*_, *F*_0_, and *F*_∞_ are used to denote the prebleach intensity, the fluorescence intensity immediately after the photobleaching, and the fluorescence intensity obtained after the recovery has plateaued, respectively (Fig 1C). When a partportion of fluorescently tagged molecules exists as an immobile pool in the ROI, only the mobile fraction (*M*_*f*_) of fluorescentce molecules will contribute to the fluorescence recovery (Fig 1C). The immobile fraction is formally defined as 1−*M*_*f*_, where *M*_*f*_ is given by

$$\begin{array}{c} M_{f}=\frac{F_{\infty }-F_{0}}{F_{i}-F_{0}}. \end{array}$$ (1)

By fitting a FRAP curve with appropriate mathematical models to describe the FRAP, the dynamics of fluorescently labeled molecules can be quantitatively analyzed in terms of kinetic parameters such as the half time of recovery (*τ*_1/2_), diffusion coefficient (*D*(*t*) or *D*), binding rate constants (*k*_*on*_ or *k*_*off*_), and mobile fractions (*M*_*f*_) [1, 8, 9]. These and other nomenclature and symbols used throughout the manuscript were are summarized in Table 1.

**Table 1:** Nomenclature and symbols.

**FRAP data**	
*F*_*rawData*_(*t*)	Raw FRAP data
*F*_*Data*_(*t*)	Back ground corrected FRAP data
*F*_0_	Florescence right after photobleaching
*F*_*i*_	Prebleach steady state fluorescence
*F*_∞_	Postbleach steady state fluorescence
*M*_*f*_	Mobile fraction
*τ*_1/2_	Half time of recovery
*F*_*rawWhole*_(*t*)	Fluorescence intensity from the whole image
*F*_*Whole*_(*t*)	Back ground corrected *F*_*rawWhole*_(*t*)
**FRAP model**	
ℝ^2^	Infinite plane
*D*	Normal diffusion coefficient
*D*(*t*)	Anomalous diffusion coefficient
Γ	Diffusion coefficient for anomalous diffusion
*α*	Anomalous diffusion exponent *D*(*t*) = Γ*t* ^*α*−1^/4
*k*_*on*_	On binding rate constant
*k*_*off*_	Off binding rate constants
*κ*	Photofading rate constant
*I*(*x*, *y*)	Gaussian laser profile
*r*_*n*_	Nominal radius
*r*_*e*_	Effective radius
*γ*	*r*_*n*_/*r*_*e*_
*q*	Quantum yield
*ϵ*	Laser attenuation constant
Φ_*D*_(*x*, *y*, *t*)	Fundamental solution of heat equation
*K*, *K*_*a*_, *K*_*l*_	Bleaching depth parameters
*F*_*AD*_(*t*)	Anomalous diffusion FRAP equation
*F*_*ND*_(*t*)	Normal diffusion FRAP equation
*F*_*b*_	Background fluorescence
*u*	Fluorescent molecule concentration
*u*_*i*_	Postbleach fluorescent molecule concentration
*τ*_*D*_, *τ*, *τ*_Γ_	Diffusion time, $\tau_{D}=r_{e}^{2}/(4D),\tau =r_{e}^{2}/4,\tau_{\Gamma }=\alpha r_{e}^{2}/\Gamma$
*u*_*t*_	∂*u*/∂*t*
Δ	Laplacian operator, ∂^2^/∂*x* ^2^+∂^2^/∂*y* ^2^
**Operators**	
*c*_1_, *c*_2_	Constants
*φ*, *ψ*	Functions
ℒ	Linear operator, ℒ(*c*_1_ *φ*+*c*_2_ *ψ*) = *c*_1_ℒ(*φ*)+*c*_2_ℒ(*ψ*)
ℒ_*ND*_	Operator for normal diffusion. ℒ_*ND*_ = ∂/∂*t*−*D*Δ
ℒ_*AD*_	Operator for anomalous diffusion. ℒ_*ND*_ = ∂/∂*t*−*D*(*t*)Δ
ℒ_*dAD*_	Operator for anomalous diffusion with photofading
ℒ_*BD*_	Operator for binding diffusion
**Subscripts**	
*ND*, *AD*	Normal Diffusion, Anomalous Diffusion
*d*	Photofading corrected
*M*_*f*_	Mobile fraction corrected
*a*	Affine scaling
*l*	Linear scaling
**Scaled FRAP equation**	
*F*(*t*)	Unscaled fluorescence intensity
*f*(*t*)	Scaled fluorescence intensity
*f*_*dAD*_(*t*)	*F*_*AD*_(*t*) corrected for photofading
*f*_*dND*_(*t*)	*F*_*ND*_(*t*) corrected for photofading
*f*_*ADaM*_*f*__(*t*)	*F*_*AD*_(*t*) corrected for mobile faction *M*_*f*_ (affine)
*f*_*NDaM*_*f*__(*t*)	*F*_*ND*_(*t*) corrected for mobile faction *M*_*f*_ (affine)
*f*_*ADlM*_*f*__(*t*)	*F*_*AD*_(*t*) corrected for mobile faction *M*_*f*_ (linear)
*f*_*NDlM*_*f*__(*t*)	*F*_*ND*_(*t*) corrected for mobile faction *M*_*f*_ (linear)
*f*_*dADaM*_*f*__(*t*)	*F*_*AD*_(*t*) corrected for photofading and *M*_*f*_ (affine)
*f*_*dNDaM*_*f*__(*t*)	*F*_*ND*_(*t*) corrected for photofading and *M*_*f*_ (affine)
*f*_*dADlM*_*f*__(*t*)	*F*_*AD*_(*t*) corrected for photofading and *M*_*f*_ (linear)
*f*_*dNDlM*_*f*__(*t*)	*F*_*ND*_(*t*) corrected for photofading and *M*_*f*_ (linear)

How to quantitatively analyze FRAP data is still an active area of research, as several different factors affect the accuracy of FRAP measurements. For example, those factors that can occur during FRAP experiments include but are not limited to diffusion of molecules during photobleaching as the result of the finite time it takes to bleach an ROI [2, 10–12], photo-switching of fluorescent proteins [13, 14], and photofading that can occur when the sample is repetitively imaged during the recovery phase [15]. To correct for these processes, various FRAP models have been developed and successfully applied in FRAP analysis. Additionally, several corrections, scalings and normalizations are typically made to FRAP data in order to apply FRAP models for quantitative FRAP analysis. First, to adjust the basal fluorescence intensity to true zero, a constant background fluorescence is subtracted from the FRAP data. Next, an additional correction has to be made to account for the loss of fluorescence due to photofading, a process that occurs as the result of repetitively imaging the specimen during the recovery phase of the experiment. FRAP data are also typically normalized to set the prebleach intensity to one in order to be able to compare data across experiments [3, 16, 17]. Last, but not least, another critical factor that must be taken into account in FRAP analysis is the possible presence of an immobile fraction (Fig 1C).

These corrections are important for a number of reasons. For example, since photofading during the recovery phase can be easily confused with an immobile fraction (Fig 1B), it is critical to distinguish photofading from an immobile fraction [3, 6, 13, 15, 18–20]. However, when corrections for both mobile fraction and photofading are made by introducing additional scalings and normalizations, FRAP models may become complicated, and even worse some FRAP models may not be compatible with a certain type of scaling. Therefore, caution must be used in FRAP analysis; otherwise errors introduced by incorrect scaling may lead to unreliable results. Moreover, since different commonly used scaling schemes generate FRAP curves with significantly different shapes (Fig 1D–1F), it is not obvious if quantitative FRAP analyses on differently scaled FRAP data should yield the same kinetic parameters. To address these issues, we here consider various scalings and normalizations commonly used to analyze FRAP data [3, 16, 19, 20] through a combination of mathematical and experimental approaches. Based on our analysis, we propose a hierarchical workflow to correctly combine necessary corrections, scalings and normalizations to perform quantitative FRAP analysis.

## Methods and materials

### FRAP data

We analyzed previously published confocal FRAP data obtained in COS-7 cells for a series of plasma membrane proteins including Flotillin-RFP (Flot-RFP), YFP-GL-GPI, mEmerald-caveolin1 (mEmeraldCav1), and Alexa488-labeled cholera toxin B-subunit (Alexa488-CTxB) [2]. A fluorescent lipid analog that incorporates into the plasma membrane, DiIC16, was also studied [2]. All of the FRAP data were collected using experimental conditions optimized for each construct using a Zeiss 510 confocal. Details regarding cell labeling and reagents, FRAP methods, and effective radius (*r*_*e*_) measurements were described in more detail elsewhere [2, 5, 11].

### Data fitting

Analysis of FRAP data was performed using previously described MATLAB programs [2, 5, 11]. For data fitting, a nonlinear least-squares fitting routine (nlinfit.m) available in MATLAB^®^ (version 7.10, R2010a, The Mathworks, Inc.) was employed.

### Photofading rate, *κ*

To determine a photofading rate, the mean fluorescence was recorded from the whole images labeled with each of the above mentioned proteins or lipid probes under the same image acquisition conditions used in the FRAP experiments. These data were averaged for at least five cells for each protein molecule and fitted to a photofading model [15, 21]

$$\begin{array}{c} F_{Whole}\left(t\right)=F_{i}e^{-\kappa t} \end{array}$$ (2)

where *κ*s^−1^ is a photofading rate.

### Statistics

All data analysis was performed using MATLAB^®^ (version 7.10, R2010a, The Mathworks, Inc.) Bar graphs represent means ± s.d. or s. e. as indicated. Statistical significance was assessed using the Student t-test.

## Theory and results

### Invariance of underlying kinetic equations under scaling or normalization

In quantitativeies FRAP analysis, parameters such as diffusion coefficients or binding rate constants that characterize the kinetics of interest are determined by comparing FRAP data with theoretical FRAP curves generated from idealized mathematical models. In many cases, the underlying mechanisms behind fluorescence recovery can be described by normal or anomalous diffusion. In mathematical diffusion models, we assume that diffusion is homogeneous in space and is unrelated to the cellular structure or underlying volume. For the purpose of this discussion we will consider the case of diffusion of proteins or lipid probes in the plasma membrane. We also assume that the bleaching spot size is much smaller than the total image so that the cell membrane can be treated as in the infinite plane (ℝ^2^). Based on these assumptions, diffusion processes in the cell membrane are approximated by normal or anomalous diffusion kinetics in the infinite plane (ℝ^2^):

$$\begin{array}{c} \left\{ \begin{array}{cc} u_{t}= & D\Delta u\\ u_{t}= & D(t)\Delta u \end{array}\right. \end{array}$$ (3)

where *D* or *D*(*t*)(*μ*m^2^/s) are diffusion coefficienents. Here, *u* is the concentration of fluorescently tagged proteins, $u_{t}=\frac{\partial u}{\partial t}$ is the partial derivative of *u* with respect to time, and $\Delta u=\frac{\partial^{2}u}{\partial x^{2}}+\frac{\partial^{2}u}{\partial y^{2}}$ is the sum of second order partial derivative of *u* with respect to space, where $\frac{\partial }{\partial t}$ and $\Delta =\frac{\partial^{2}}{\partial x^{2}}+\frac{\partial^{2}}{\partial y^{2}}$ are called differentiation operators. A linear combination of differentiation operators (i.e. sum of differentiation operators with coefficients) is called a differential operator. Therefore, normal and anomalous diffusion in Eq 3 can be represented in terms of differential operators ℒ_*ND*_ and ℒ_*AD*_ as

$$\begin{array}{c} \left\{ \begin{array}{cc} {𝓛}_{ND}= & \frac{\partial }{\partial t}-D\Delta \\ {𝓛}_{AD}= & \frac{\partial }{\partial t}-D\left(t\right)\Delta \end{array}.\right. \end{array}$$ (4)

If *u* is a solution to a normal or anomalous diffusion equations in ℝ^2^ (Eq 3) then ℒ_*ND*_(*u*) = 0 or ℒ_*AD*_(*u*) = 0 because

$$\begin{array}{ccc} 0 & = & \frac{\partial u}{\partial t}-D\left(t\right)\Delta u\\ & = & (\frac{\partial }{\partial t}-D\left(t\right)\Delta )u\\ & = & {𝓛}_{AD}\left(u\right). \end{array}$$ (5)

An operator ℒ is called linear if ℒ satisfies

$$\begin{array}{c} 𝓛\left(c_{1}\varphi +c_{2}\psi \right)=c_{1}𝓛\left(\varphi \right)+c_{2}𝓛\left(\psi \right) \end{array}$$ (6)

where *c*_1_ and *c*_2_ are constants and *φ* and *ψ* are real valued differentiable functions. Another important property of differential operators is that the image of constants under differential operator is zero. Additionally, a differential operator ℒ satisfies

$$\begin{array}{c} 𝓛(c)=0, \end{array}$$ (7)

for a constant *c*, because the derivative of a constant is zero.

Many different mathematical models for anomalous diffusion are available [22]. Here, we only consider an anomalous diffusion model due to a time dependent time dependent coefficient *D*(*t*) for simplicity. Within this framework, a normal diffusion equation or ℒ_*ND*_ can be regarded as a special case of the anomalous diffusion equation or ℒ_*AD*_ where *D*(*t*) = *D*, a constant independent of time. Therefore it is enough to investigate the properties of ℒ_*AD*_ to study those of ℒ_*ND*_ and ℒ_*AD*_ (Eq 5).

In mathematical sciences, an affine transformation of a function *φ* is of the form *φ* ↦ *c*_1_ *φ*+*c*_2_, where *c*_1_ (≠ 0) and *c*_2_ are real numbers (*c*_1_, *c*_2_ ∈ ℝ). In particular, a special case of an affine transformation for *c*_2_ = 0, *φ* ↦ *c*_1_ *φ* is called a linear transformation. For a given operator or kinetic equation, if a transformation or scaling of a solution such as an affine transformation or a linear transformation is still the solution of the same kinetic equation, we say the kinetic equation is invariant under the scaling, the scaling is valid for the kinetic equation, or the scaling is compatible with the kinetic equation.

In a FRAP analysis, it is important to verify the scaling invariance of the underlying kinetic equations in order to guarantee that the exactly same kinetic parameter values are obtained before and after scaling of FRAP data or FRAP equations. By direct calculation, it can be shown that both normal and anomalous diffusion equations are affine scaling invariant. For this, notice that ℒ_*AD*_ and ℒ_*ND*_ are linear differential operators that satisfy

$$\begin{array}{c} {𝓛}_{AD/AD}\left(c_{1}u+c_{2}\right)=c_{1}{𝓛}_{AD/AD}\left(u\right)+{𝓛}_{AD/AD}\left(c_{2}\right)=0 \end{array}$$ (8)

from Eqs 6 and 7 if *u* is a solution of normal or anomalous diffusion equation (Eq 3). This further indicates that linear scaling is also compatible with normal and anomalous diffusion equations.

Not all differential operators are invariant under affine scaling. For example, the operator ℒ_*dAD*_ for a photofading diffusion equation is not compatible with affine scaling. If diffusion occurs in the presence of photofading during image acquisition, then the fluorescent molecule concentration *u* satisfies the photofading diffusion equation,

$$\begin{array}{c} \left\{ \begin{array}{cc} u_{t}= & D\Delta u-\kappa u\\ u_{t}= & D(t)\Delta u-\kappa u \end{array}\right. \end{array}$$ (9)

Note that these photofading diffusion equations can be represented by the linear operator ℒ_*dAD*_ = ∂/∂*t*−*D*(*t*)Δ+*κ* or ℒ_*dND*_ = ∂/∂*t*−*D*Δ+*κ*. ℒ_*dAD*_ and ℒ_*dND*_ are not invariant under affine scaling because, for the solution *u* for the photofading diffusion equation (Eq 9) and constants *c*_1_ and *c*_2_

$$\begin{array}{ccc} {𝓛}_{dAD}\left(c_{1}u+c_{2}\right) & = & (\frac{\partial }{\partial t}-D\left(t\right)\Delta +\kappa )\left(c_{1}u+c_{2}\right)\\ & = & c_{1}(\frac{\partial u}{\partial t}-D\left(t\right)\Delta u+\kappa u)+\kappa c_{2}\\ & = & \kappa c_{2} \end{array}$$ (10)

where *κc*_2_ ≠ 0. However, since ℒ_*dAD*_(*c*_1_ *u*) = 0, linear scaling is still compatible with the photofading diffusion equation. The list of operators that appeared here are summarized in Table 1.

### FRAP equations for normal and anomalous diffusion

We previously reported FRAP equations that can be specifically used to analyze confocal FRAP data. The first formalism represents an extension of the Axelrod FRAP equation [3] for recovery by free diffusion into a circular bleaching spot. It is modified to account for diffusion that can occur due to the finite time required to bleach using laser scanning confocal microscopes [2, 11]. The second extends this to the case of anomalous diffusion [23]. Here, we summarize the confocal FRAP equations for both free diffusion and anomalous diffusion.

If we assume that the bleaching spot size is small compared with the cell size, then we can treat the cell as an infinite plane, ℝ^2^[2, 5, 11]. In the infinite plane, we also assume the photobleaching laser intensity profile is described as a Gaussian laser [2, 5, 11]:

$$\begin{array}{c} I\left(x,y\right)=\frac{2I_{0}}{\pi r_{n}^{2}}\exp(-\frac{2(x^{2}+y^{2})}{r_{n}^{2}}) \end{array}$$ (11)

Here, *I*_0_ is the total laser intensity and *r*_*n*_ is defined as a nominal radius of the bleaching spot, which is identical to the radius of the circular ROI. Notice that ∬_ℝ^2^_ *I*(*x*, *y*)*dxdy* = *I*_0_. During the recovery phase, the intensity of the photobleaching laser is attenuated, which can be represented as *ϵI*(*x*, *y*), where *ϵ* is the attenuation factor (*ϵ* ≪ 1). Since the fluorescence intensity is directly proportional to both the excitation laser intensity *ϵI*(*x*, *y*) and fluorescent molecule concentrations, if we let the fluorescent molecule concentration at the location (*x*, *y*) at time *t* be *u*(*x*, *y*, *t*), then the fluorescence intensity is described by

$$\begin{array}{c} F(x,y,t)=q\epsilon I(x,y)u(x,y,t) \end{array}$$ (12)

where the proportionality constant *q* is called the fluorescence quantum yield. Therefore, the fluorescence intensity from a circular bleaching spot, or region of interest (ROI) is obtained by integrating the spatial fluorescence intensity over the ROI

$$\begin{array}{c} F\left(t\right)=\iint_{{\mathbb{R}}^{2}}q\epsilon I\left(x,y\right)u\left(x,y,t\right)dxdy \end{array}$$ (13)

If the fluorescent species is diffusive (normal or anomalous), then the transport can be described by the diffusion equation (Eq 3). As demonstrated in previous studies [2], the initial condition for Eq 3 is given by a postbleach profile

$$\begin{array}{c} u\left(x,y,0\right)=u_{i}(1-K\exp(-\frac{2(x^{2}+y^{2})}{r_{n^{2}}})) \end{array}$$ (14)

where *u*_*i*_ is the prebleach fluorescent concentration, and *r*_*e*_ is the half width at the approximately 14% of bleaching depth from the top. We define *r*_*e*_ as the effective radius of a postbleach profile, in contrast to the nominal radius (*r*_*n*_) from a user-defined bleaching spot radius. The effective radius *r*_*e*_ was introduced to correct for diffusion during the photobleach [11]. To solve the diffusion equations (Eq 3) for a given initial condistion (a postbleach profile, Eq 14), if we introduce a new time scale $s=\int_{0}^{t}D(\bar{t})d\bar{t}$, then in a new time scale *s*, the left hand side (LHS) and right hand side (RHS) of Eq 3 become

$$\begin{array}{ccc} LHS & = & \frac{\partial }{\partial t}\left(u\right)\\ & = & \frac{\partial }{\partial s}\left(u\right)\frac{\partial s}{\partial t}\\ & = & \frac{\partial }{\partial s}\left(u\right)D\left(t\right)\\ RHS & = & D(s)\Delta u \end{array}$$ (15)

which yields

$$\begin{array}{c} \frac{\partial u}{\partial s}=\Delta u. \end{array}$$ (16)

Notice that Eq 16 corresponds to the pure diffusion equation when *D* = 1 (Eq 3) while the time independent initial condition remains the same (Eq 14). Now, Eq 16 can be solved by applying standard textbook techniques for *D* = 1 as

$$\begin{array}{ccc} u\left(x,y,t\right) & = & \Phi_{D}\left(x,y,t\right)*u\left(x,y,0\right)\\ & \equiv & \iint_{{\mathbb{R}}^{2}}\Phi_{D}\left(x-\bar{x},y-\bar{y},t\right)u\left(\bar{x},\bar{y},0\right)d\bar{x}d\bar{y}\\ \Phi_{D}\left(x,y,t\right) & = & \frac{1}{4\pi Dt}e^{-\frac{x^{2}+y^{2}}{4Dt}} \end{array}$$ (17)

where Φ_*D*_(*x*, *y*, *t*) is called the fundamental solution of diffusion equation.

If this solution for *u*(*x*, *y*, *t*) is used in the FRAP equation (Eq 13), then the integral can be simplified as

$$\begin{array}{ccc} F(s) & = & F_{i}\;(1-\frac{K}{1+\gamma^{2}+2s/\tau }) \end{array}$$ (18)

where $\tau =r_{e}^{2}/s$, *γ* = *r*_*n*_/*r*_*e*_, and $K=\left(1-\frac{F_{0}}{F_{i}}\right)(1+\gamma^{2})$. In particular, for $D(t)=\frac{1}{4}\Gamma t^{\alpha -1}$[23], $s=\frac{1}{4\alpha }\Gamma t^{\alpha }$. By substituting back, the FRAP equations in the original time scale *t* is

$$\begin{array}{ccc} F_{AD}\left(t\right) & = & F_{i}\;(1-\frac{K}{1+\gamma^{2}+2t^{\alpha }/\tau_{\Gamma }}) \end{array}$$ (19)

where $\tau_{\Gamma }=\alpha r_{e}^{2}/\Gamma$. On the other hand, for normal diffusion kinetics for *D*(*t*) = *D*, the FRAP equation simplifies to

$$\begin{array}{ccc} F_{ND}\left(t\right) & = & F_{i}\;(1-\frac{K}{1+\gamma^{2}+2t/\tau_{D}}) \end{array}$$ (20)

where $\tau_{D}=r_{e}^{2}/(4D)$. The meaning of subscripts that appeared here awere summarized in Table 1.

### Correcting raw FRAP data for background fluorescence

Another important assumption in fluorescence microscopy is that the concentration of fluorescently labeled molecules is directly proportional to the fluorescence intensity, which requires the fluorescence intensity goes to zero when no fluorescent molecules are present. This is also an important hypothesis for mathematical models for diffusion FRAP. In our previous experiments [2], the background fluorescence level wais 4%-10% (6.9±2.9, *n* = 17) of maximal fluorescence level in most cases (Fig 1B). To adjust to true zero fluorescence, the by background fluorescence needs to be subtracted from the FRAP data. If we let *F*_*rawData*_(*t*) and *F*_*rawWhole*_(*t*) represent the raw fluorescence signals from an ROI and and the whole image, and *F*_*b*_ represent the background fluorescence directly measured from experiments, then the background corrected FRAP data, *F*_*Data*_(*t*) and background corrected whole image fluorescence, *F*_*Whole*_(*t*) are given by

$$\begin{array}{c} \left\{ \begin{array}{cc} F_{Data}\left(t\right)= & F_{rawData}\left(t\right)-F_{b}\\ F_{Whole}\left(t\right)= & F_{rawWhole}\left(t\right)-F_{b} \end{array}.\right. \end{array}$$ (21)

From a mathematical perspective, this represents an affine scaling of the form *f*(*t*) = *c*_1_ *F*(*t*)+*c*_2_, where *c*_1_ = 1 and *c*_2_ = −*F*_*b*_. Background correction is important in FRAP analysis, in the sense that this step aligns the FRAP data to theoretical FRAP models, in which zero fluorophore concentration is assumed to correspond to zero fluorescence intensity. Without background correction, FRAP data do not satisfy FRAP equations, and compability of scalings and normalizations of FRAP data or FRAP equations do not make sense. Therefore, background fluorescence has to be corrected before scalings and normalization are applied.

#### Validation of normalizing FRAP data from a 0–1 scale and normalizing FRAP data to prebleach intensity

After background fluorescence is corrected, various scaling and normalization schemes can follow in many different ways depending on the kinetics of interest. FRAP data are commonly normalized from raw fluorescent intensity units into a 0–1 scale [24, 25]. Specifically, the fluorescence data *F*_*Data*_(*t*) with *F*_*Data*_(0) = *F*_0_ and *F*_*Data*_(∞) = *F*_∞_ can be normalized into 0–1 scale as (Fig 1E),

$$\begin{array}{ccc} f(t) & = & \frac{F_{Data}\left(t\right)-F_{0}}{F_{i}-F_{0}}\\ & = & \left(\frac{1}{F_{i}-F_{0}})\;F_{Data}\left(t\right)-(\frac{F_{0}}{F_{i}-F_{0}}\right) \end{array}$$ (22)

which also represents an affine scaling, *f*(*t*) = *c*_1_ *F*(*t*)+*c*_2_, where *c*_1_ = 1/(*F*_*i*_−*F*_0_) and *c*_2_ = −*F*_0_/(*F*_*i*_−*F*_0_). Note that *f*(0) = 0 and *f*(*t* < 0) = 1 (Fig 1E).

FRAP data are also often normalized by the prebleach steady state fluorescence intensity (*F*_*i*_) i.e., *F*(*t*)/*F*_*i*_ (Fig 1F). This sets the prebleach fluorescence intensity to one and thus allows for the direct comparison of datasets obtained from different samples, which otherwise may differ in absolute fluorescence intensity [11, 26]. When a FRAP curve is normalized by the prebleach steady state fluorescence intensity (*F*_*i*_), i.e.

$$\begin{array}{c} f\left(t\right)=\frac{F_{Data}\left(t\right)}{F_{i}} \end{array}$$ (23)

then the postbleach steady state, *f*(∞) may be less than 1 depending on the presence of an immobile fraction (Fig 1F). From a mathematical perspective, this is an example of a linear scaling, which has the form *cF*(*t*) for some nonzero constant *c*. In this particular case, *c* = 1/*F*_*i*_

In principle, these corrections and scalings can be applied to FRAP data or to FRAP equations or even to the both. To justify these three different approaches in diffusion FRAP analysis, we let *v* = *c*_1_ *u*+*c*_2_, and *c*_1_ ≠ 0, where the unscaled variable *u*(*x*, *y*,0) satisfies the diffusion equations (Eq 3). Since the diffusion equations are described by an affine scaling invariant linear differential operator ℒ_*ND*_ or ℒ_*AD*_, the scaled variable *v* also satisfies the same diffusion equation as *u* by Eq 5. Therefore, *f*_*v*_(*t*), a corresponding FRAP equation for a scaled variable *v* can be derived by integrating *v* over the ROI (Eq 13);

$$\begin{array}{ccc} f_{v}\left(t\right) & = & \iint_{{\mathbb{R}}^{2}}q\epsilon I\left(x,y\right)v\left(x,y,t\right)dxdy\\ & = & \;\iint_{{\mathbb{R}}^{2}}q\epsilon I(x,y)(c_{1}u+c_{2})dxdy\\ & = & c_{1}\iint_{{\mathbb{R}}^{2}}q\epsilon I\left(x,y\right)u\left(x,y,t\right)dxdy+c_{2}\iint_{{\mathbb{R}}^{2}}q\epsilon I\left(x,y\right)dxdy\;\\ & = & c_{1}F_{u}\left(t\right)+c_{2}F_{i}. \end{array}$$ (24)

This indicates that affine scaling of FRAP equations can be directly interpreted as the same affine scaling of a solution to the diffusion equation (Eq 3), which is invariant under affine scaling. Consequently, affine scaling of FRAP equations, *c*_1_ *F*(*t*)+*c*_2_ and the unscaled FRAP equation, *F*(*t*) share the same diffusion kinetic properties (i.e. the same *D*; Eq 3). This additionally demonstrates that different affine scalings of diffusion FRAP data share the same diffusion kinetic properties. i.e. if *F*_*Data*_(*t*) ≃ *F*(*t*) then *c*_1_ *F*_*Data*_(*t*)+*c*_2_ ≃ *c*_1_ *F*(*t*)+*c*_2_where *F*(*t*) and *c*_1_ *F*(*t*)+*c*_2_ share the same kinetic properties.

To experimentally confirm this conclusion, FRAP data for Alexa488-CTxB were background corrected and transformed using affine (Fig 1E) or linear (Fig 1F) scaling schemes. Then, *D* was calculated by fitting the scaled FRAP data with *F*_*ND*_(*t*) (Eq 20). Indeed, exactly the same diffusion coefficient, *D* = 0.2 *μ*m^2^/s, was obtained from the differently scaled FRAP data (Fig 1E and 1F). This verifies that as expected, either converting FRAP data to a 0–1 scale using an affine scaling scheme or adjusting the prebleach intensity values of FRAP data to one using a linear scaling scheme yields the same fitting results.

### Correction for photofading in diffusion FRAP analysis: Photofading Model

As demonstrated in Fig 1B, a significant loss of fluorescence may occur even under low excitation intensity. The rate of fluorescence loss as the result of observing the sample during the recovery phase, which we refer to as photofading, can be quantified from the fluorescence intensity from the whole image, or *F*_*Whole*_(*t*) (Eq 21).

To investigate and understand the possible role of photofading during image acquisition in quantitative FRAP analysis from a theoretical perspective, we assume photofading is a 1^st^ order decay process of fluorophores with a photofading rate *κ*. Then, the fluorophore concentration *u* satisfies

$$\begin{array}{c} \frac{d}{dt}u\left(x,y,t\right)=-\kappa u\left(x,y,t\right), \end{array}$$ (25)

and the solution of which is given by

$$\begin{array}{c} u\left(x,y,t\right)=u\left(x,y,0\right)e^{-\kappa t} \end{array}$$ (26)

with a constant initial condition, *u*(*x*, *y*,0) = *u*_*i*_.

Having derived the solution for a photofading model (Eq 26), we next assess the effect of photofading in the ROI due to image acquisition by integrating the solution as (Eq 13);

$$\begin{array}{ccc} F\left(t\right) & = & \iint_{{\mathbb{R}}^{2}}q\epsilon I\left(x,y\right)(u\left(x,y,0\right)e^{-\kappa t})dxdy\\ & = & e^{-\kappa t}F_{i} \end{array}$$ (27)

where *F*_*i*_ = ∬_ℝ^2^_ *qϵI*(*x*, *y*)*dxdy* is the initial fluorescence intensity. As long as the bleaching spot size is small enough, then the total number of fluorescent molecules will remain relatively constant even after the photobleaching event if the fluorescence intensity is measured over a large domain such as the whole image (Fig 1B). Under these conditions, any decrease in fluorescence intensity over the whole image would be from photofading due to image acquisition. In addition, since Eq 27 does not depend on the size of ROI (*r*_*n*_ in *I*(*x*, *y*) from Eq 11), the photofading rate *κ* can be determined by fitting the fluorescence from the whole image, *F*_*Whole*_(*t*)with Eq 27:

$$\begin{array}{ccc} F_{Whole}\left(t\right) & ≃ & e^{-\kappa t}F_{i}. \end{array}$$ (28)

Later on, we will use Eq 28 to validate the correction for photofading by normalizing the FRAP data by the whole image fluorescence.

**Fig 2:**
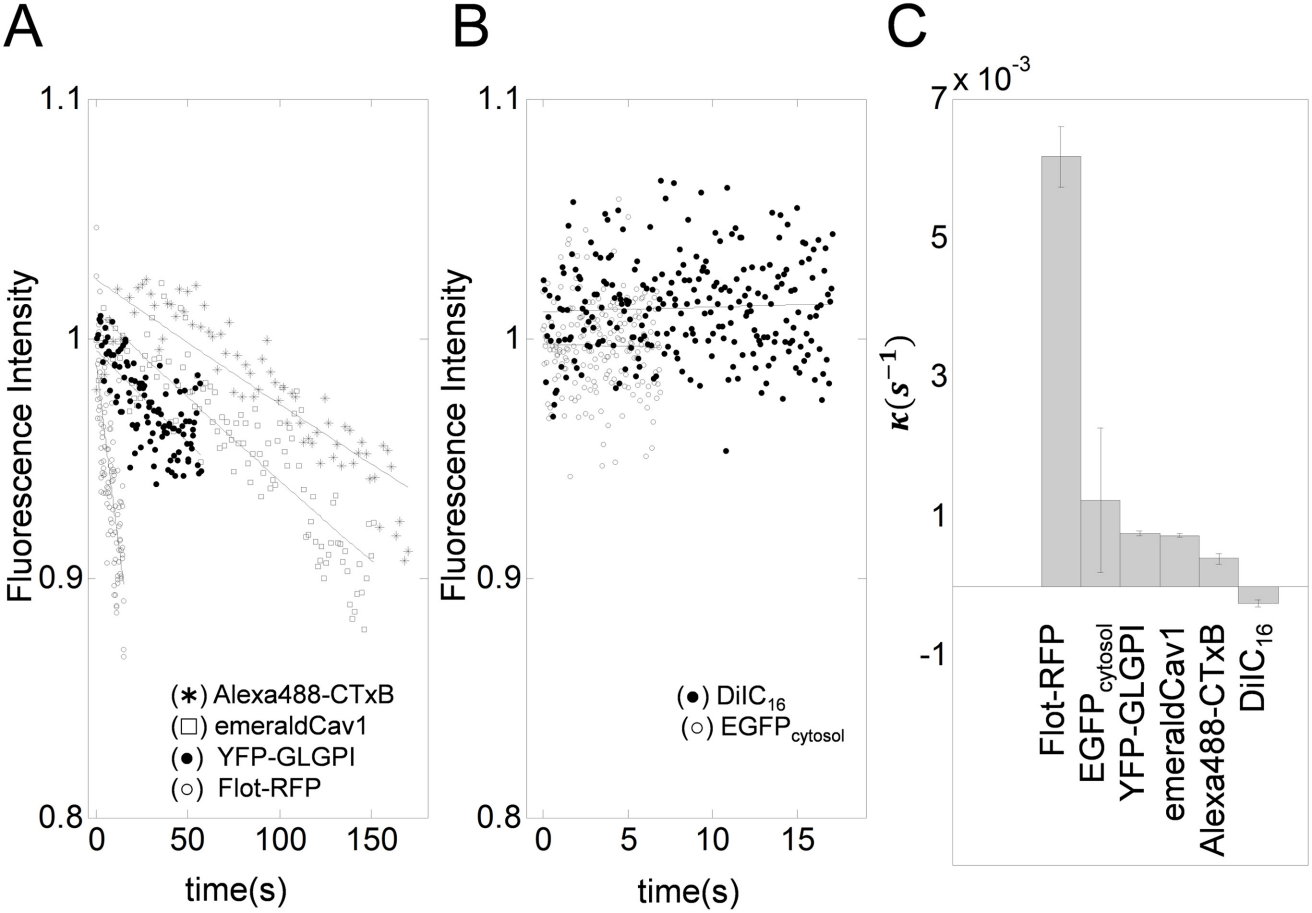
Representative photofading rates obtained from the whole image fluorescence for a variety of different fluorescently tagged molecules. A photofading model, *f*(*t*) = *e* ^−*κt*^ was fitted to normalized whole image fluorescence data *F*_*Data*_(*t*)/*F*_*i*_ averaged over multiple data sets (*n* = 10) for each of the indicated fluorescent proteins or fluorescent lipid probes. (A–B) Best fitting photofading model applied to whole image fluorescence. Solid lines show the best fitting curves. (C) Photofading rate constants obtained from the fits shown in A and B are shown in descending order. Error bar represents the standard deviation (*n* = 14).

To estimate the order of photofading rates (*κ*) observed under typical experimental conditions, we analyzed FRAP data for a series of proteins and lipids previously collected using confocal FRAP [2]. Fluorescently tagged molecules considered were Alexa488-CTxB, YFP-GLGLPI, EGFP, mEmerald-Cav1, Flot-RFP, and DiIC16, which have diffusion coefficients ranging from 0.1 ˜ 40 *μ*m^2^/s. These FRAP datasets were obtained using different filter sets and laser excitation optimized for each fluorescent molecule, and also were obtained using different time intervals to allow for differences in the kinetics of recovery. In all these cases, photofading during image acquisition could be well described by an exponential decay photofading model with *κ* values between 10^−3^ ∼ 10^−2^/s (Eq 28) as shown in Fig 2A and 2B.

### Correction of photofading by an exponential scaling of the FRAP equation

Since both diffusion and photofading occur at the same time during FRAP experiments, we next develop a FRAP diffusion model that includes a photofading component. As recent studies have described methods to correct for photofading in reaction diffusion kinetics [15], we will confine our attention to FRAP analysis for normal diffusion [2] or anomalous diffusion kinetics [19, 25]. In a recent study, Wu et al. [15] made the argument that bleaching only occurs during relatively short times (during image acquisition) and thus occurs at fixed time intervals whereas diffusion is continuous. However, for simplicity, we follow the approach of Mueller et al. [27] which assumes that both photofading and diffusion occur continuously. If diffusion occurs in the presence of photofading during image acquisition, then the fluorescent molecule concentration *u* satisfies Eq 9. To find the solution, we multiply an integration factor *e* ^*κt*^ on both sides to get

$$\begin{array}{c} \frac{\partial }{\partial t}(e^{\kappa t}u)=D\left(t\right)\Delta (e^{\kappa t}u) \end{array}$$ (29)

Next, we introduce a new variable *v* = *e* ^*κt*^ *u*. Since the exponentially scaled new variable *v* satisfies the normal or anomalous diffusion equation (Eq 3), *v*_*t*_ = *D*(*t*)Δ*v*, the solution for *v* can be found by introducing a new time scale $s=\int_{0}^{t}D(\bar{t})d\bar{t}$ as in Eqs 15–17:

$$\begin{array}{ccc} v(x,y,s) & = & e^{\kappa t(s)}u\left(x,y,s\right)\\ & = & e^{\kappa t(s)}\Phi_{D}\left(x,y,s\right)*u\left(x,y,0\right) \end{array}$$ (30)

where Φ_*D*_(*x*, *y*, *s*) is the fundamental solution of the diffusion equation for *D* = 1 (Eq 17). In a similar manner as in Eq 18, we can derive a FRAP equation in a new time scale *s* as

$$\begin{array}{c} f\left(s\right)=F_{i}e^{-\kappa t(s)}(1-\frac{K}{1+\gamma^{2}+2s/\tau }). \end{array}$$ (31)

For $D(t)=\frac{1}{4}\Gamma t^{\alpha -1}$[19, 23],

$$\begin{array}{ccc} f_{dAD}\left(t\right) & = & F_{i}e^{-\frac{\kappa \Gamma }{4\alpha }t^{\alpha }}(1-\frac{K}{1+\gamma^{2}+2t^{\alpha }/\tau_{\Gamma }}). \end{array}$$ (32)

where $\tau_{\Gamma }=\alpha r_{e}^{2}/\Gamma$. Note that, without photofading, i.e. *κ* = 0, the corrected Feder’s equation (Eq 19) is obtained. Also for normal diffusion, we choose *D*(*t*) = *D* to get

$$\begin{array}{c} f_{dND}\left(t\right)=F_{i}e^{-\kappa t}(1-\frac{K}{1+\gamma^{2}+2t/\tau_{D}}). \end{array}$$ (33)

These equations can then be used to directly fit FRAP data, *F*_*Data*_(*t*) that have previously been background corrected using Eq 21. Interestingly, Eqs 32 and 33 indicate that the photofading component and diffusion component can be easily separated from *f*_*dAD*_(*t*) and *f*_*dND*_(*t*) as

$$\begin{array}{c} \left\{ \begin{array}{cc} f_{dAD}\left(t\right)=e^{-\kappa t}F_{AD}\left(t\right) & \Leftrightarrow \frac{f_{dAD}\left(t\right)}{e^{-\kappa t}}=F_{AD}\left(t\right)\\ f_{dND}\left(t\right)=e^{-\kappa t}F_{ND}\left(t\right) & \Leftrightarrow \frac{f_{dND}\left(t\right)}{e^{-\kappa t}}=F_{ND}\left(t\right) \end{array}\right. \end{array}$$ (34)

where *F*_*AD*_(*t*) and *F*_*ND*_(*t*) are as in Eqs 19 and 20.

### Correction of photofading by an exponential scaling of FRAP data

As photofading typically manifests as an exponential decay of fluorescence over time [8, 15], photofading in FRAP data can be corrected by introducing an exponential scaling (*e* ^−*κt*^), which can either be incorporated into the FRAP equation (*e* ^−*κt*^ *F*(*t*)) or used to correct the FRAP data itself. In the latter case, the rate of photobleaching is often estimated by measuring the overall loss of fluorescence within the specimen over time during the experiment (Eq 28).

In many studies [4, 26, 28], photofading during image acquisition was corrected by dividing the FRAP data by the fluorescence intensity of the whole image at each time point:

$$\begin{array}{c} \frac{F_{Data}\left(t\right)}{F_{Whole}\left(t\right)}. \end{array}$$ (35)

Notice that fluorescence from the whole image, *F*_*Whole*_(*t*) can be described as an exponential decay (Eq 28). Also, since *F*_*Data*_(*t*) contains both diffusion and photofading components, *F*_*Data*_(*t*) can be described by *f*_*dND*_(*t*) or *F*_*dAD*_(*t*) (Eqs 32 and 33). Therefore, FRAP data from normally diffusive molecules with photofading scaled by fluorescence from the whole image can be described by a normal diffusion FRAP equation

$$\begin{array}{ccc} \frac{F_{Data}\left(t\right)}{F_{Whole}\left(t\right)} & ≃ & \left(1-\frac{K}{1+\gamma^{2}+2t/\tau_{D}}\right) \end{array}$$ (36)

which is identical to *F*_*ND*_(*t*) (Eq 20). This indicates that after correcting for photofading during image acquisition by dividing the FRAP data by the fluorescence intensity of whole image at each time point (i.e. an exponential scaling of FRAP data), the exponentially scaled FRAP data, *F*_*Data*_(*t*)/*F*_*Whole*_(*t*) can be analyzed by a pure diffusion FRAP model, which is invariant under linear and affine scalings.

### Compatibility of linear and affine scaling with exponential scaling for photofading correction

Eq 36 suggests the exponentially scaled FRAP data, *F*_*Data*_(*t*)/*F*_*Whole*_(*t*) satisfy diffusion FRAP kinetics without photofading (Eqs 19 and 20), which are compatible with both affine and linear scaling schemes. Therefore, *F*_*Data*_(*t*)/*F*_*Whole*_(*t*) can be fitted to either affine or linear scaling of diffusion FRAP equations, *F*_*AD*_(*t*) and *F*_*ND*_(*t*).

On the other hand, a photofading diffusion operator ℒ_*dAD*_ = ∂/∂*t*−*D*(*t*)Δ+*κ* is not compatible with affine scaling as shown in Eq 10. This further indicates that if exponential and affine scalings are to be used together, exponential scaling for photofading should precede any affine scaling. In contrast, since the photofading diffusion equation is invariant under a linear scaling, i.e. ℒ_*dAD*_(*cu*) = 0 for a constant *c*, exponential and linear scalings can be used together in any order.

### Correction of FRAP equations for an immobile fraction by affine or linear scaling

Another typical example of scaling for a FRAP equation is the inclusion of a mobile fraction (*M*_*f*_) [3, 9, 29]. This is typically included in the form of an affine scaling. For example, in previous study [2], we reported a diffusion FRAP model with mobile fraction *M*_*f*_ as

$$\begin{array}{c} \left\{ \begin{array}{cc} f_{NDaM_{f}}\left(t\right)= & F_{i}(1-\frac{K_{a}}{1+\gamma^{2}+2t/\tau_{D}})\;M_{f}+\left(1-M_{f}\right)F_{0}\\ f_{ADaM_{f}}\left(t\right)= & F_{i}(1-\frac{K_{a}}{1+\gamma^{2}+2t^{\alpha }/\tau_{\Gamma }})\;M_{f}+\left(1-M_{f}\right)F_{0} \end{array}.\right. \end{array}$$ (37)

Here, *K*_*a*_ = (1−*F*_0_/*F*_*i*_)(1+*γ* ^2^), which can be obtained by solving *f*_*NDaM*_*f*__(0) = *F*_0_ or *f*_*ADaM*_*f*__(0) = *F*_0_. Here the subscript *a* is used to indicate the affine scaling (Table 1). We can easily verify that this is another example of an affine scaling because *f*_*NDaM*_*f*_/*ADaM*_*f*__(*t*) = *c*_1_ *F*_*ND*/*AD*_(*t*)+*c*_2_ where *c*_1_ = *F*_*i*_ *M*_*f*_ and *c*_2_ = (1−*M*_*f*_)*F*_0_.

Since an affine scaling scheme is not compatible with an exponential scaling of the FRAP equation corrected for photofading, here, we propose and justify a new linear scaling scheme for mobile fractions, which is compatible with an exponentially scaled FRAP equation for photofading in any order:

$$\begin{array}{c} \left\{ \begin{array}{cc} f_{NDlM_{f}}\left(t\right)= & F_{\infty }\;(1-\frac{K_{l}}{1+\gamma^{2}+2t/\tau_{D}})\\ f_{ADlM_{f}}\;\left(t\right)= & F_{\infty }(1-\frac{K_{l}}{1+\gamma^{2}+2t^{\alpha }/\tau_{\Gamma }}) \end{array}.\right. \end{array}$$ (38)

where *K*_*l*_ = (1−*F*_0_/*F*_∞_)(1+*γ* ^2^), which can be obtained by solving *f*_*NDlM*_*f*__(0) = *F*_0_ or *f*_*ADlM*_*f*__(0) = *F*_0_. Here, the subscript *l* is used to indicate the linear scaling (Table 1).

We next justify *f*_*ADlM*_*f*__(*t*) and *f*_*NDlM*_*f*__(*t*) by showing *f*_*NDaM*_*f*__(*t*) = *f*_*NDlM*_*f*__(*t*) for normal diffusion. In the anomalous diffusion case, *f*_*ADaM*_*f*__(*t*) = *f*_*ADlM*_*f*__(*t*) can be shown in the exactly same way. To see *f*_*NDaM*_*f*__(*t*) = *f*_*NDlM*_*f*__(*t*), note first that *F*_*i*_ *M*_*f*_+(1−*M*_*f*_)*F*_0_ = *F*_∞_ from Eq 1

$$\begin{array}{ccc} M_{f}\left(F_{i}-F_{0}\right) & = & F_{\infty }-F_{0}\\ F_{i}M_{f}+\left(1-M_{f}\right)F_{0} & = & F_{\infty }. \end{array}$$ (39)

Therefore,

$$\begin{array}{ccc} & & f_{NDaM_{f}}\left(t\right)-f_{NDlM_{f}}\left(t\right)\\ & = & F_{i}\;(1-\frac{K_{a}}{1+\gamma^{2}+2t/\tau_{D}})\;M_{f}+\left(1-M_{f}\right)F_{0}-F_{\infty }\;(1-\frac{K_{l}}{1+\gamma^{2}+2t/\tau_{D}})\\ & = & F_{i}M_{f}+\left(1-M_{f}\right)F_{0}-F_{\infty }+\frac{F_{\infty }K_{a}-F_{i}K_{l}}{1+\gamma^{2}+2t/\tau_{D}}\\ & = & \frac{F_{\infty }K_{a}-F_{i}K_{l}}{1+\gamma^{2}+2t/\tau_{D}}\\ & = & \frac{1+\gamma^{2}}{1+\gamma^{2}+2t/\tau_{D}}(F_{\infty }\;[1-\frac{F_{0}}{F_{\infty }}]-F_{i}\;[1-\frac{F_{0}}{F_{i}}]\cdot [\frac{F_{\infty }-F_{0}}{F_{i}-F_{0}}])\\ & = & 0 \end{array}$$ (40)

This proves that both *f*_*NDaM*_*f*__(*t*) and *f*_*NDlM*_*f*__(*t*) are identical scalings (i.e. *f*_*NDaM*_*f*__(*t*) = *f*_*NDlM*_*f*__(*t*)) for diffusion FRAP equations corrected for an immobile fraction. Thus, so are *f*_*ADaM*_*f*__(*t*) and *f*_*ADlM*_*f*__(*t*) for anomalous diffusion. Since only a linear scaling is compatible with a exponentially scaled FRAP equation corrected for photofading, *f*_*ADlM*_*f*__(*t*) or *f*_*NDlM*_*f*__(*t*) can be used with an exponential scaling of a FRAP equation corrected for photofading (Eqs 32 and 33) in any scaling order.

### FRAP model corrected for both photofading and an immobile fraction

Because the photofading diffusion equations are invariant under linear scaling i.e. ℒ_*dAD*_(*cu*) = 0 or ℒ_*dND*_(*cu*) = 0, it is possible to derive a valid FRAP equation corrected for both photofading and an immobile fraction:

$$\begin{array}{c} \left\{ \begin{array}{cc} f_{dNDlM_{f}}\left(t\right)= & F_{\infty }e^{-\kappa t}\;(1-\frac{K_{l}}{1+\gamma^{2}+2t/\tau_{D}})\\ f_{dADlM_{f}}\;\left(t\right)= & F_{\infty }e^{-\kappa t}(1-\frac{K_{l}}{1+\gamma^{2}+2t^{\alpha }/\tau_{\Gamma }}) \end{array}.\right. \end{array}$$ (41)

Eq 41 demonstrates that correction for photofading and a mobile fraction can be done by multiplying a correction factor for photofading, *e* ^−*κt*^ and a correction factor for the mobile fraction *F*_∞_ to the ideal diffusion FRAP equations, *F*_*ND*_(*t*) or *F*_*AD*_(*t*). Since multiplication is commutative, this further confirms that exponential and affine scalings can be used together in any order, i.e. *F*_∞_{*e* ^−*κt*^ *F*_*AD*_(*t*)} = *e* ^−*κt*^{*F*_∞_ *F*_*AD*_(*t*)}.

From Eq 41, it is also possible to derive a diffusion FRAP equation corrected for photofading and a mobile fraction by an affine scaling as *f*_*dNDlM*_*f*__(*t*) = *e* ^−*κt*^{*f*_*NDaMf*_(*t*)}

$$\begin{array}{c} \left\{ \begin{array}{cc} f_{dNDaM_{f}}\left(t\right)= & e^{-\kappa t}(F_{i}\;(1-\frac{K_{a}}{1+\gamma^{2}+2t/\tau_{D}})M_{f}+\left(1-M_{f}\right)F_{0})\\ f_{dADaM_{f}}\left(t\right)= & e^{-\kappa t}(F_{i}\;(1-\frac{K_{a}}{1+\gamma^{2}+2t^{\alpha }/\tau_{\Gamma }})M_{f}+\left(1-M_{f}\right)F_{0}) \end{array},\right. \end{array}$$ (42)

where where we used the fact, *f*_*NDaMf*_(*t*) = *f*_*NDlMf*_(*t*):

$$\begin{array}{ccc} f_{dNDlM_{f}}\left(t\right) & =F_{\infty }\{e^{-\kappa t}F_{ND}\left(t\right)\}=e^{-\kappa t}\{F_{\infty }F_{ND}\left(t\right)\}\\ & =e^{-\kappa t}\{f_{NDaMf}\left(t\right)\}=e^{-\kappa t}\{f_{NDlMf}\left(t\right)\} & =f_{dNDaM_{f}}\left(t\right) \end{array}$$ (43)

The same holds for *f*_*dADaM*_*f*__(*t*) = *e* ^−*κt*^{*f*_*ADaM*_*f*__(*t*)}. Again, the meanings of the subscripts are explained in Table 1.

### Importance of correct sequence of scalings and applying scaling correctly

**Fig 3:**
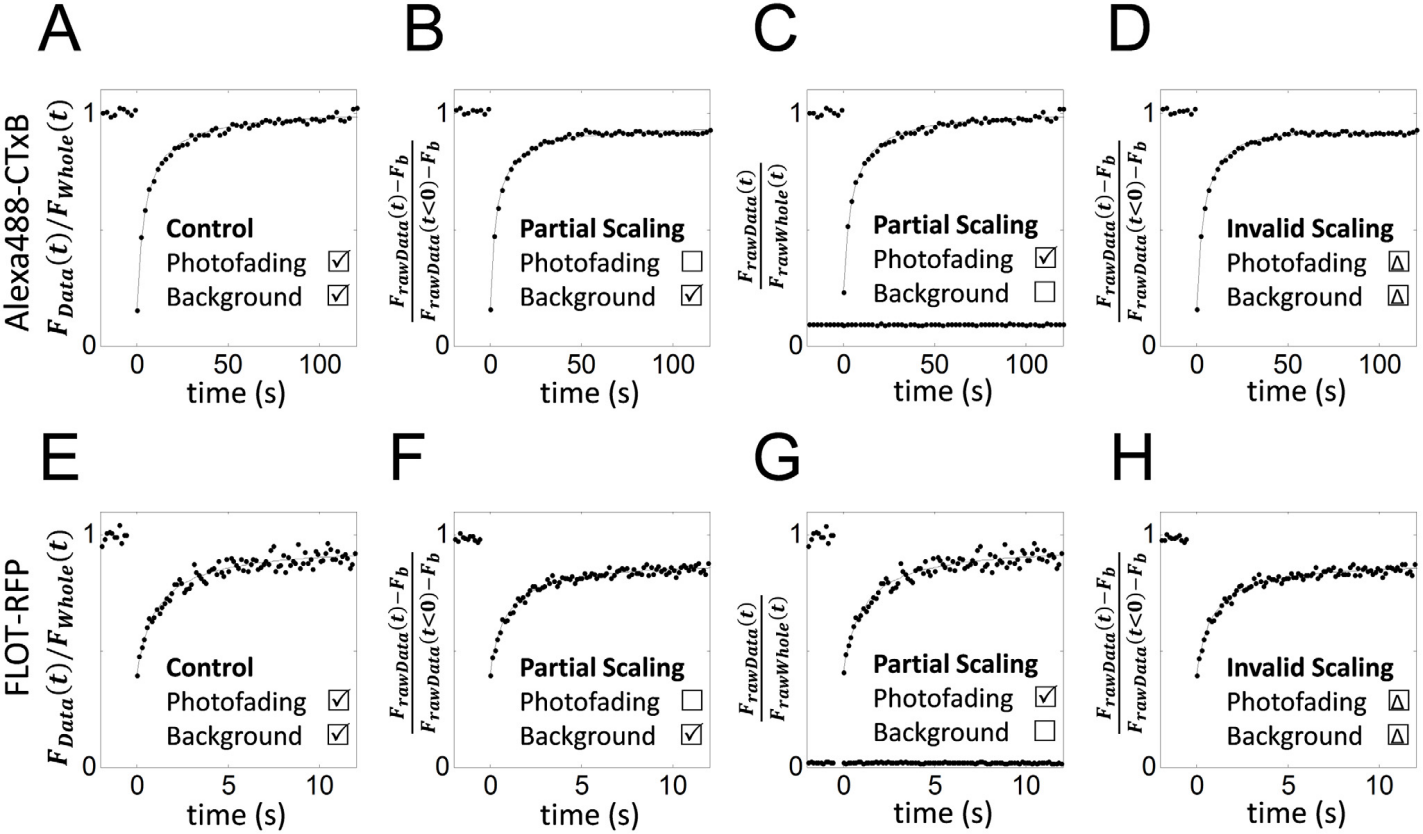
Errors caused by incorrect sequence of scaling or by applying partial scaling corrections. FRAP analysis was performed on Alexa488-CTxB FRAP data (A–D) or FLOT-RFP FRAP data (E–H) using different scaling scenarios. (A,E) As a positive control, FRAP data corrected for both background fluorescence and photofading were analyzed using *f*_*NDaM*_*f*__(*t*) (Eq 37) in the correct order. (B,F) Background-corrected FRAP data were analyzed by *f*_*NDaM*_*f*__(*t*) (Eq 37), ignoring the contribution of photofading. (C,G) Photofading-corrected FRAP data, *F*_*Data*_(*t*)/*F*_*Whole*_(*t*) were analyzed by *f*_*NDaM*_*f*__(*t*) (Eq 37), ignoring any background correction. Lower lines show background fluorescence, *F*_*b*_. (D,H) Background corrected FRAP data were analyzed using a photofading and mobile fraction corrected FRAP equation obtained by using an incorrect scaling order (Eq 44). Dots (•) show the mean FRAP data averaged over one set of experiments (*n* = 12 and 13 for FLOT-RFP and Alexa488-CTxB, respectively) and solid lines show the best fitting curve.

**Fig 4:**
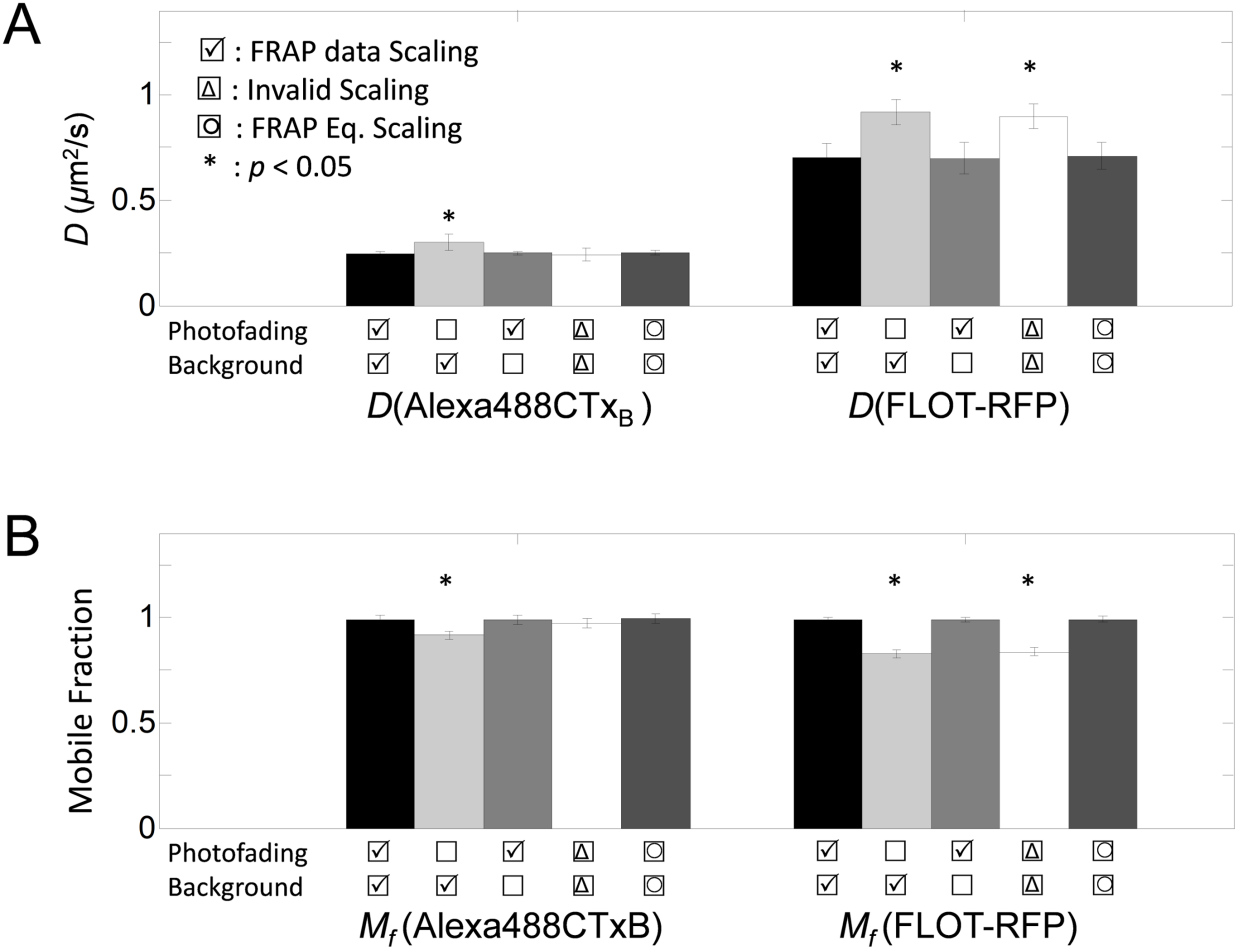
Best fitting diffusion coefficients (*D*) and mobile fractions (*M*_*f*_) of Alexa488-CTxB and FLOT-RFP under different scaling scenarios. (A) and (B) show the best fitting diffusion coefficients (*D*), mobile fraction (*M*_*f*_), and standard errors (*N* = 4 × 12 or *N* = 3 × 13). Statistically significant differences are denoted with asterisk (*).

To investigate the importance of performing the right scaling in the correct sequence in FRAP analysis, FRAP data for FLOT-RFP (*n* = 12) and Alexa488-CTxB (*n* = 13) were analyzed under different scaling assumptions. The choice of these proteins is based on the fact that FLOT-RFP showed much a higher photofading rate than Alexa488-CTxB (Fig 2), even though the diffusion coefficients of these proteins are of a similar order of magnitude [30]. As shown in Fig 3, we compared the effects of only partially correcting the FRAP data or analyzing it using incorrect scaling. First, we correctly analyzed the data. Here, the FRAP data were corrected for both background (Eq 21) and photofading *F*_*Data*_(*t*)/*F*_*Whole*_(*t*). The fully corrected FRAP data were analyzed using *f*_*NDaM*_*f*__ (t) (Eq 37) (Fig 3A and 3E). *D* and *M*_*f*_ values from the fits are shown in Fig 4A and 4B respectively. Next, we examined the effect of failing to account for photofading. In this case the FRAP data were only corrected for background using *F*_*Data*_(*t*)/*F*_*Data*_(*t* < 0). The background-corrected data were then fitted using *f*_*NDaM*_*f*__(*t*)(Eq 37) to obtain *D* and *M*_*f*_ (Fig 3B and 3F). We also assessed the effect of not including a background correction. Here the data were only corrected for photofading using *F*_*rawData*_(*t*)/*F*_*rawWhole*_(*t*) (Eq 21). The photofading-corrected FRAP data were then fit by *f*_*NDaM*_*f*__(*t*) (Eq 37) (Fig 3C and 3G). Finally, we performed FRAP analysis using an incorrect scaling sequence. For this analysis the background corrected FRAP data, *F*_*Data*_(*t*) was fitted with an incorrectly scaled FRAP equation (cf: *f*_*dNDaM*_*f*__(*t*) in Eq 42)

$$\begin{array}{c} f\left(t\right)=F_{i}e^{-\kappa t}\;(1-\frac{K_{a}}{1+\gamma^{2}+2t/\tau_{D}})M_{f}+\left(1-M_{f}\right)F_{0} \end{array}$$ (44)

The effects of either partially correcting the FRAP data or performing FRAP analysis using an incorrectly scaled FRAP equation differed somewhat (Fig 4). When only background correction was performed (no photofading correction), *D* and *M*_*f*_ were significantly different from those obtained for the correctly analyzed dataset (Student t-test, *p* < 0.05). Similarly, FRAP analysis by invalid FRAP scaling yielded significantly different *D* and *M*_*f*_ for FLOT-RFP (Fig 4). However, when only the photofading correction was included (no background correction), the resulting *D* and *M*_*f*_ were not significantly different than *D* and *M*_*f*_ from the correctly analyzed FRAP curves for either FLOT-RFP or Alexa488-CTxB (Fig 4).

Not surprisingly, *M*_*f*_ calculated from datasets where the FRAP data were corrected for photofading (*F*_*rawData*_(*t*)/*F*_*rawWhole*_(*t*)) was higher than that of the normalized FRAP data with no photofading correction (*F*_*Data*_(*t*)) as shown in Fig 4. This is presumably because of the scaling *e* ^*κt*^ ≃ 1/*F*_*Whole*_(*t*) > 1 for *t* > 0, which leads to

$$\begin{array}{ccc} F_{Data}\left(t\right)/F_{Whole}\left(t\right) & ≃ & e^{\kappa t}F_{Data}\left(t\right)\\ & \geq & F_{Data}\left(t\right) \end{array}$$ (45)

from Eqs 28 and 36. Moreover, the FRAP data with higher photofading rate (FLOT-RFP) showed a larger difference in *M*_*f*_ before and after correction for photofading than the FRAP data with the lower photofading rate (Alexa488-CTxB) (Fig 4). Similarly, when the FRAP data were not corrected for photofading, faster diffusion coefficients were measured (Fig 4).

In principle, scaling to correct for photofading and mobile fractions can be applied to either FRAP data or the FRAP equation and both can be mathematically justified. We experimentally tested this by also fitting the data using a photofading-corrected FRAP equation (Eq 42). As expected, analyses using photofading-corrected FRAP data or a photofading-corrected FRAP equation yielded very similar *D* and *M*_*f*_ values (Fig 4). This indicates that either approach is equally valid for correcting for photofading.

### Magnitude of errors caused by incorrect sequence of scalings or by applying incompatible scaling

**Fig 5:**
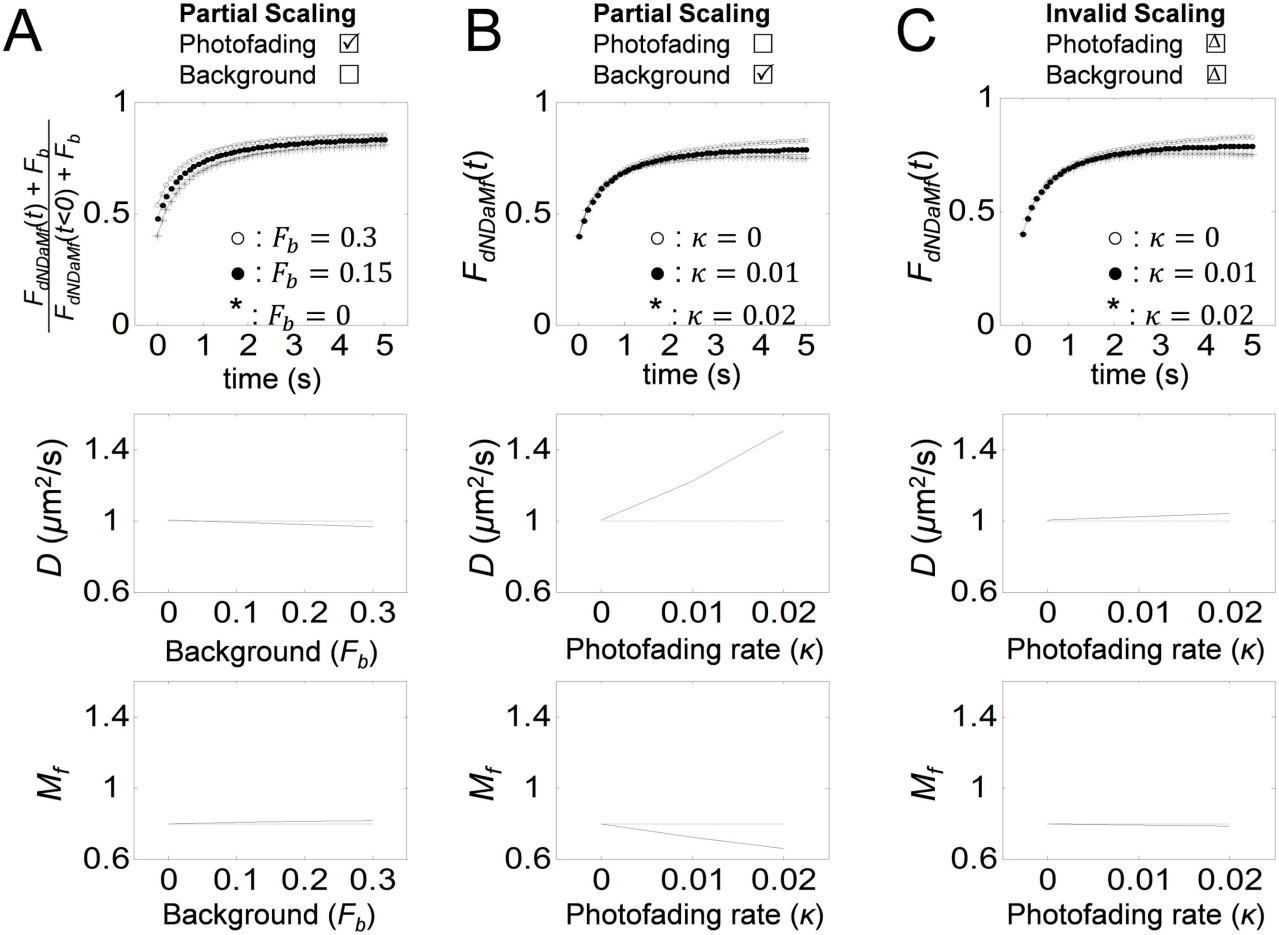
Analysis of the magnitude of errors caused by applying incompatible scaling or by using an incorrect sequence of scaling during FRAP analysis. FRAP curves were simulated assuming *D* = 1*μ*m^2^/s, *F*_0_ = 0.4, *F*_*i*_ = 1, *r*_*n*_ = 1*μ*m, and *r*_*e*_ = 2*μ*m under different scaling scenarios. Curves were then analyzed as indicated to obtain the best fits for *D* and *M*_*f*_. (A) FRAP curves were simulated using (*f*_*dNDaM*_*f*__(*t*)+*F*_*b*_)/(*f*_*dNDaM*_*f*__(*t* < 0)+*F*_*b*_) assuming *κ* = 0.005 s^−1^ and 0 ≤ *F*_*b*_ ≤ 0.3 and then analyzed using *f*_*dNDaM*_*f*__(*t*) (Eq 42) while ignoring *F*_*b*_. (B) FRAP curves were simulated using *f*_*dNDaM*_*f*__(*t*) assuming *M*_*f*_ = 0.8 and 0 ≤ *κ* ≤ 0.02 s^−1^ and then analyzed using *f*_*NDaM*_*f*__(*t*) (Eq 37) while ignoring photofading. (C) FRAP curves were simulated using *f*_*dNDaM*_*f*__(*t*) (Eq 42) assuming *M*_*f*_ = 0.8 and 0 ≤ *κ* ≤ 0.02 s^−1^ and analyzed using an incorrectly scaled FRAP equation (Eq 44). Panels in the 2nd row show best fitting *D* as a function of variable conditions, *F*_*b*_ and *κ*. Panels in the 3rd row show best fitting *M*_*f*_ as a function of variable conditions, *F*_*b*_ and *κ*.

We next further looked into the magnitude of errors caused either by not correcting for background fluorescence and photofading or by applying an incorrect sequence of scaling in terms of diffusion coefficients and mobile fractions using simulated FRAP curves. First of all, to estimate the magnitude of errors caused by not correcting for background fluorescence, FRAP curves were generated using *f*_*dNDaM*_*f*__(*t*)+*F*_*b*_ (Eq 42) for different levels of background fluorescence, 0 ≤ *F*_*b*_ ≤ 0.3. This covers a range of background fluorescence level we observed experimentally of 4%–10% (6.9±2.9, *n* = 17). The normalized simulated curves (*f*_*dNDaM*_*f*__(*t*)+*F*_*b*_)/(*f*_*dNDaM*_*f*__(*t* < 0)+*F*_*b*_) were fitted to *f*_*dNDaM*_*f*__(*t*) (Eq 42) to obtain *D* and *M*_*f*_ under conditions where background fluorescence was not accounted for (Fig 5A). This omission did not have a significant effect on either *M*_*f*_ or *D*. This result supports the observation that not correcting for background fluorescence did not substantially alter the results of the FRAP analysis for Alexa488-CTxB and FLOT-RFP (Fig 4). This is probably because even though *F*_*b*_ was between 4% and 10% of the maximal fluorescence level in our experiments, the normalization step, (*f*_*dNDaM*_*f*__(*t*)+*F*_*b*_)/(*f*_*dNDaM*_*f*__(*t* < 0)+*F*_*b*_) reduces this difference to the order of 1%.

Errors in *D* and *M*_*f*_ can also be introduced by not correcting for photofading. To examine the magnitude of such errors, FRAP curves were generated using *f*_*dNDaM*_*f*__(*t*) (Eq 42) for different levels of photofading, 0 ≤ *κ* ≤ 0.02. The simulated curves were fitted to *f*_*NDaM*_*f*__(*t*) (Eq 37) to obtain the best fitting *D* and *M*_*f*_. In contrast to the background correction, the photofading correction had a significant impact on both *D* and *M*_*f*_ (Fig 5B). For a higher photofading rate, larger *D* and smaller *M*_*f*_ were obtained. Since some fluorescent proteins may have *κ* as high as 6 × 10^−3^ (Fig 2C), the magnitude of errors introduced by not correcting for photofading can reach as much as 20%.

Finally, to estimate the magnitude of errors obtained when an incorrect sequence of scaling is applied, FRAP curves were generated by *f*_*dNDaM*_*f*__(*t*) (Eq 42) for different levels of photofading 0 ≤ *κ* ≤ 0.02. The simulated curves were fitted for *D* and *M*_*f*_ using an incorrectly scaled FRAP equation (Eq 44). Although exponential scaling is not compatible with affine scaling, the errors caused by applying an incorrect sequence of scaling were not as large as the errors obtained when photofading was ignored (Fig 5C). However, it is worth mentioning that the small error caused by an incorrect scaling sequence here is from a single example. Therefore, it is still desirable to use a valid scaling scheme.

Among the scaling scenarios considered here, the magnitudes of errors were largest when photofading was not corrected and smallest when background was not corrected. The wrong scaling order (invalid scaling) yielded an intermediate level of errors. Overall, these results match very well with the results obtained from the analysis of the Alexa488-CTxB and FLOT-RFP FRAP data (Fig 4).

### Photofading can be confused with anomalous diffusion

**Fig 6:**
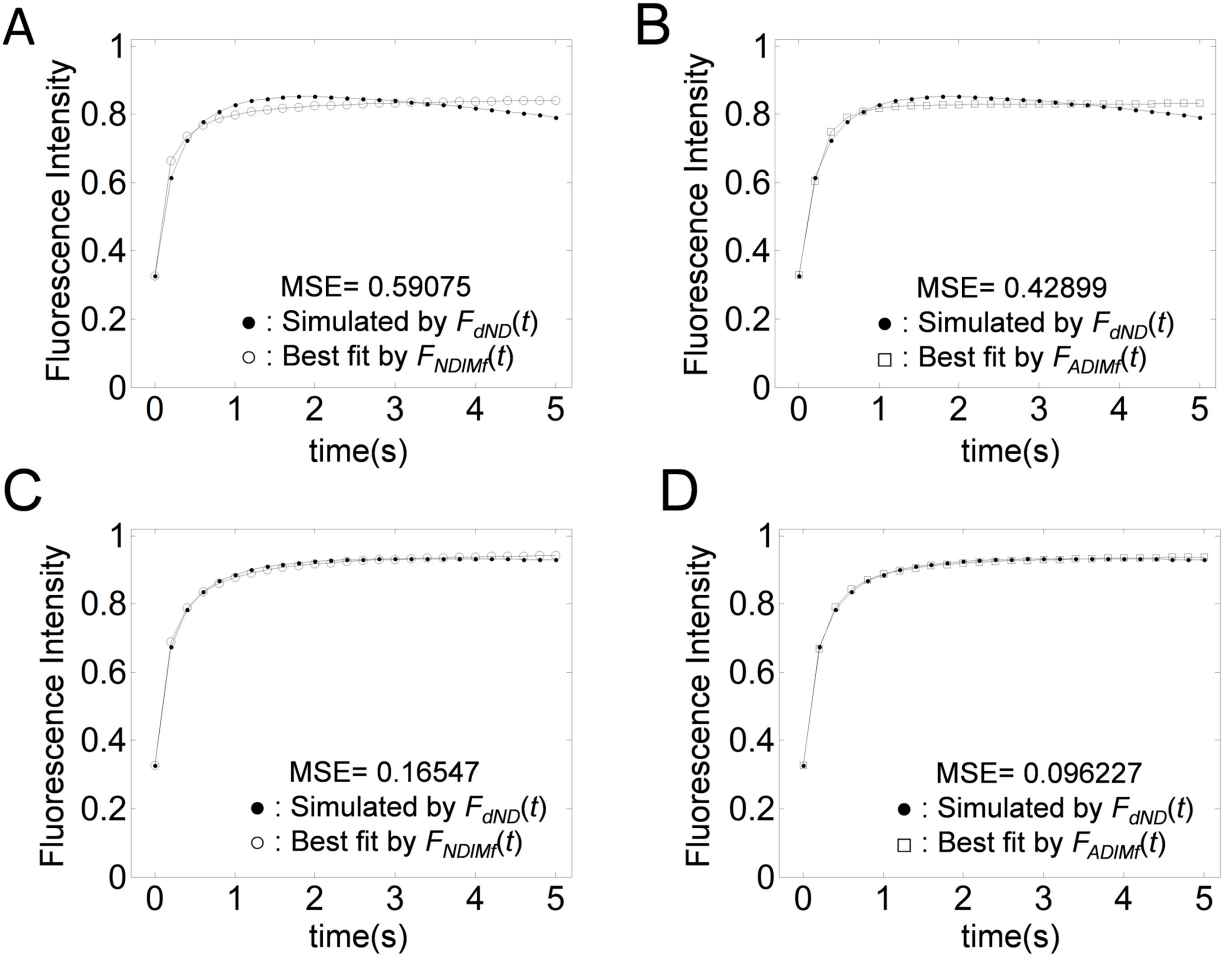
Effect of photofading during image acquisition on fits to FRAP curves for normal diffusion versus anomalous diffusion. FRAP data with photofading (•) were simulated using *f*_*dADaM*_*f*__(*t*) (Eq 42) assuming either a photofading rate of *κ* = 0.04 s^−1^ (A,B) or *κ* = 0.01 s^−1^ (C,D) under the conditions *D* = 1*μ*m^2^/s, *F*_0_ = 0.2, *M*_*f*_ = 0.8, *r*_*n*_ = 0.2 *μ*m, and *r*_*e*_ = 1 *μ*m. The simulated curve was next fitted by *f*_*NDaM*_*f*__(*t*) (Eq 37) (A,C) or *f*_*NDaM*_*f*__(*t*) (Eq 37) (B,D). The resulting best fitting parameters were *D* = 2.1*μ*m^2^/s and *M*_*f*_ = 0.83 in (A), $\frac{1}{4}\Gamma =9.33$ and *M*_*f*_ = 0.83 in (B), *D* = 2.7*μ*m^2^/s and *M*_*f*_ = 0.95 in (C), and $\frac{1}{4}\Gamma =5.32$ and *M*_*f*_ = 0.94 in (D).

To study the effect of photofading on diffusion FRAP analysis further, a FRAP curve generated by *f*_*dAD*_(*t*) (Eq 32), was fitted either with a pure diffusion FRAP equation with an immobile fraction, *f*_*NDaM*_*f*__(*t*) (Eq 37) or the corrected Feder’s Equation with an immobile fraction, *f*_*ADaM*_*f*__(*t*) (Eq 37)). For a high photofading rate (*κ* = 0.04/s), the FRAP data decrease after reaching the postbleach steady state. In this case, neither model fits the FRAP data successfully (Fig 6A and 6B). Under mild photofading conditions (*κ* = 0.01/s), the corrected Feder’s equation provides a better fit than the pure diffusion FRAP equation (Fig 6C and 6D). This signifies that (i) correction for photofading is required for quantitative FRAP analysis with high accuracy, and (ii) without correction for photofading during image acquisition, even FRAP data from pure diffusion can potentially be mistaken as demonstrating anomalous diffusion. Therefore, to avoid this type of complexity, it is required to correct for photofading before scaling for *M*_*f*_.

### Hierarchical workflow of diffusion FRAP analysis

**Fig 7:**
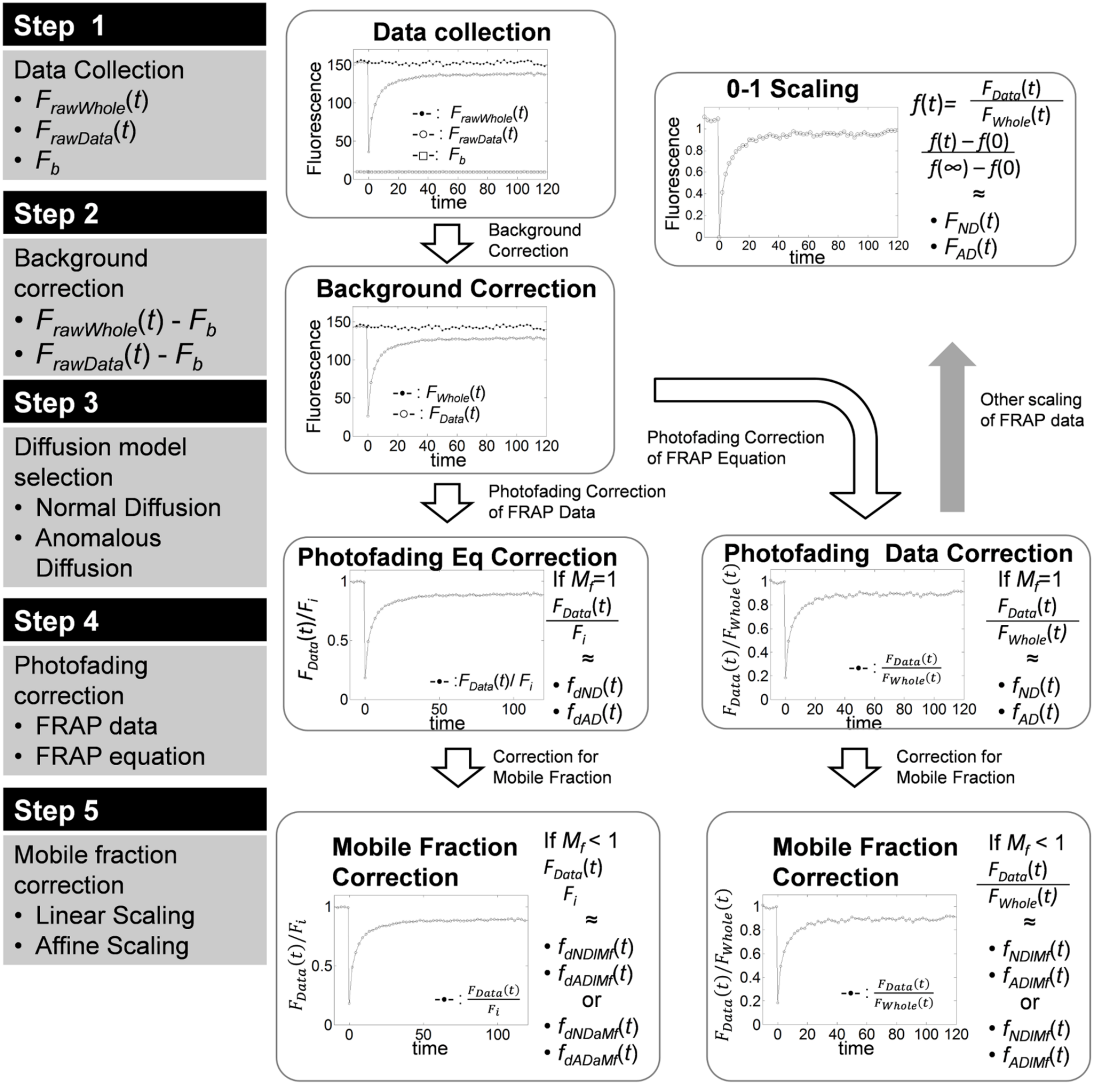
Hierarchical workflow of diffusion FRAP analysis to correct for background, photofading and an immobile fraction.

Since scaling for photofading and mobile fraction are not always interchangeable, and photofading can be confused with anomalous diffusion, here we propose a hierarchical workflow for diffusion FRAP analysis (Fig 7) to systematically avoid compatability issues or possible confusion. For FRAP analysis, three fluorescence datasets have to be collected: 1) FRAP data from the bleach ROI, 2) whole image fluorescence, and 3) background fluorescence (Step 1). Background fluorescence should be removed from the fluorescence data (Eq 21) in order to apply any FRAP model and prevent possible overestimation of *M*_*f*_ (Step 2). For diffusion FRAP analysis, either a normal diffusion FRAP equation or an anomalous diffusion FRAP equation should be selected depending on the kinetic property of the molecules of interest (Step 3). Once a model is chosen, a correction for photofading has to be considered (Step 4). The photofading correction can be performed either by scaling the FRAP data by whole image fluorescence (Eqs 36 and 36), or by scaling the FRAP equation exponentially by multiplying the FRAP equation (Eq 42) by the exponential decaying factor *e* ^−*κt*^. In the latter case, the photofading rate *κ* should be determined by fitting Eq 2 to the whole image fluorescence (*F*_*Whole*_(*t*)) after correcting for background (Eq 21). Also, to make the FRAP data and FRAP equation comparable, either the FRAP equations or the FRAP data have to be scaled. To scale the FRAP equation, *F*_*i*_ can be chosen to be the prebleach steady state fluorescence intensity of the FRAP data. However, in most cases the FRAP data are normalized by *F*_*Data*_(*t*)/*F*_*i*_ to be fitted by FRAP equations. Since the correction of the FRAP data does not require an extra step to determine the photofading rate *κ*, scaling of the FRAP data itself may be a simpler option. If *D* is the parameter of interest, photofading corrected FRAP data can be further scaled to 0–1 by an affine transform (*f*(*t*)−*f*(0))/(*f*(∞)−*f*(0)) (Fig 7, gray arrow). Otherwise, a mobile fraction correction should also be considered (Step 5). To correct the FRAP data or FRAP equations for a mobile fraction, either linear (Eq 41) or affine (Eq 42) scaling of FRAP equations are available (Step 5). While exponential and linear scaling can be used together in any order, exponential scaling must precede affine scaling to be valid. In either case, since exponential scaling for photofading can be done first, correction for photofading is recommended before mobile fraction correction.

## Discussion

Normalizations and scaling are indispensable components in FRAP analysis to account for either inherent properties of the fluorescently labeled molecules of interest (e.g. mobile fraction), or photochemical properties of the fluorescent tag (e.g. photofading during image acquisition). In general, scalings that are widely used in FRAP analysis fall into three mathematical categories: (i) exponential scaling, (ii) affine scaling, and (iii) linear scaling. Since several of these scalings should be combined to account for various factors, verifying the compatibilities of these scalings is important to guarantee the accuracy of FRAP analysis. In the current study, we investigated how to best combine these various scalings with pure diffusion and anomalous diffusion FRAP equations to appropriately correct FRAP data for background, photofading and immobile fractions. We showed that both the affine and linear scalings are compatible with diffusion FRAP equations using the property of linear differential operators.

Multiple affine and linear scalings can also be combined. To see this, let *φ*(*u*) = *c*_1_ *u*+*c*_2_ and *ψ*(*u*) = *c*_3_ *u*+*c*_4_; then the combination (or composition of two scaling functions) is

$$\begin{array}{ccc} \psi \circ \varphi (u) & = & \psi (\varphi (u))\\ & = & \left(c_{1}c_{3}\right)u+\left(c_{2}c_{3}+c_{4}\right) \end{array}$$ (46)

which is another affine scaling. Since diffusion equations are invariant under affine scalings, any combinations of multiple affine and linear scalings may be used with either diffusion FRAP data or diffusion FRAP equations. For example, if we consider a linear normalization (*F*(*t*)/*F*_*i*_), followed by affine scaling for mobile fraction correction (Eq 37), then we obtain another affine scaling in the form of

$$\begin{array}{c} \frac{M_{f}}{F_{i}}F\left(t\right)+\left(1-M_{f}\right)F_{0} \end{array}$$ (47)

which is a compatible scaling with diffusion equations.

As part of this study, we considered how to combine corrections for photofading with other scaling factors and normalizations. For example, a linear scaling *v* = *cu* where *u* satisfies a diffusion equation was compatible with diffusion-photofading equation (Eq 9). Therefore, if we combine a linear normalization (*F*(*t*)/*F*_*i*_) with another linear scaling for the mobile fraction (*F*_∞_ *F*(*t*)) followed by exponential scaling to correct for photofading then we get

$$\begin{array}{c} \frac{F_{\infty }}{F_{i}}e^{-\kappa t}F\left(t\right) \end{array}$$ (48)

which also satisfies the diffusion-photofading equation and is therefore valid. On the other hand, an affine scaling of the solution to a diffusion-photofading equation does not satisfy the diffusion-photofading equation. Therefore, affine scalings cannot be used in an arbitrary order in combination with the exponential scaling of a FRAP equation to correct for photofading. Importantly, when exponential scaling is applied to diffusion FRAP data by dividing by the whole image fluorescence (*F*_*Data*_(*t*)/*F*_*Whole*_(*t*)), the scaled diffusion FRAP data satisfies the diffusion equations, and therefore is compatible with both the affine and linear scalings. The compatibility of these various scalings is summarized in Table 2.

**Table 2:** Diffusion FRAP models corrected for photofading and immobile fraction and their compatibilities. In Binding Diffusion* (14), *c*_*k*_ represents the concentration of ligand receptor complex in the *k*th image, $c_{k}^{M}$ is the mobile fraction of *c*, *β* is the immobile fraction, and *τ* is the time needed for each image scan. Finally, *g*_1_ and *g*_2_ are the bleaching functions for bounded (*c*) and free (*u*) proteins.

**Underlying Kinetics**	**Photofading**	**Immobile Fraction**	**FRAP data/FRAP equation**	**Compatibility**
Free Diffusion	Scaling of FRAP Equation	Linear Scaling	$\frac{F_{Data}(t)}{F_{i}}\;\underset{\text{Fit}}{⇐}\;f_{dNDlM_{f}}(t)$ (Eq 41)	Yes
Free Diffusion	Scaling of FRAP Equation	Affine Scaling	$\frac{F_{Data}(t)}{F_{i}}\;\underset{\text{Fit}}{⇐}\;f_{dNDaM_{f}}(t)$ (Eq 42)	No: Scaling order matters
Free Diffusion	Scaling of FRAP Data	Linear Scaling	$\frac{F_{Data}(t)}{F_{whole}(t)}\;\underset{\text{Fit}}{⇐}\;f_{NDlM_{f}}(t)$ (Eq 38)	Yes
Free Diffusion	Scaling of FRAP Data	Affine Scaling	$\frac{F_{Data}(t)}{F_{whole}(t)}\;\underset{\text{Fit}}{⇐}\;f_{NDaM_{f}}(t)$ (Eq 37)	Yes
Anomalous Diffusion	Scaling of FRAP Equation	Linear Scaling	$\frac{F_{Data}(t)}{F_{i}}\;\underset{\text{Fit}}{⇐}\;f_{dADlM_{f}}(t)$ (Eq 41)	Yes
Anomalous Diffusion	Scaling of FRAP Equation	Affine Scaling	$\frac{F_{Data}(t)}{F_{i}}\;\underset{\text{Fit}}{⇐}\;f_{dADaM_{f}}(t)$ (Eq 42)	No: Scaling order matters
Anomalous Diffusion	Scaling of FRAP Data	Linear Scaling	$\frac{F_{Data}(t)}{F_{whole}(t)}\;\underset{\text{Fit}}{⇐}\;f_{ADlM_{f}}(t)$ (Eq 38)	Yes
Anomalous Diffusion	Scaling of FRAP Data	Affine Scaling	$\frac{F_{Data}(t)}{F_{whole}(t)}\;\underset{\text{Fit}}{⇐}\;f_{ADaM_{f}}(t)$ (Eq 37)	Yes
Binding Diffusion	Scaling of FRAP Equation	Linear Scaling	$\begin{array}{c} \frac{F_{Data}(t)}{F_{i}}\;\underset{\text{Fit}}{⇐}\;\frac{c_{2}^{M}}{c_{1}}=\delta (1-\beta )\\ \;\frac{c_{k}^{M}}{c_{1}}=(1-\beta )\left(g_{2}^{k-1}-\left[g_{2}^{k-1}-\frac{c_{k-1}^{M}}{c_{1}(1-\beta )}\right]e^{-\tau k_{off}}\right)g_{1} \end{array}$	Yes
Binding Diffusion	Scaling of FRAP Equation	Affine Scaling	NA	No: Regardless of Scaling Order

Correcting for immobile fractions and photofading also has an important role in quantitative FRAP analysis. Without correcting for an immobile fraction, diffusion FRAP equations cannot be fitted properly to the theoretical model for pure diffusion, since the diffusion FRAP equations assume 100% recovery. This could have important consequences for more complicated types of analysis, as incorrectly fitted pure diffusion data could potentially be confused with other kinetics such as binding kinetics and/or kinetics of two different population of proteins [29, 31]. Similarly, without correcting for photofading, diffusion FRAP analysis may yield erroneous mobile fractions or diffusion coefficients (Figs 3 and 4). If photofading is ignored, FRAP data from free diffusion kinetics could also be mistakenly identified as anomalous diffusion, a form of diffusion with a time dependent diffusion coefficient [19, 32]. Finally, we note that recently, Wu et al. [15] studied the effects of scaling for photofading in reaction-type FRAP analysis and obtained a similar conclusion about the importance of correcting for photofading as presented in this work. In their study, Wu et al. considered binding kinetics without diffusion as

$$\begin{array}{c} \left\{ \begin{array}{cc} \frac{du}{dt}= & -\lambda u\\ \frac{dc}{dt}= & k_{on}u-k_{off}c\\ u(0)= & u_{1},\;c\left(0\right)=\delta c_{1} \end{array}\right. \end{array}$$ (49)

where *c* and *u* are the concentration of ligand receptor complex and soluble cytosolic ligand, with initial concentration level *c*_1_ and *u*_1_, respectively. Here, *λ* and *δ* represent the photofading rate and a parameter describing the amount of photobleached molecules due to photobleaching. Based on this model, the authors concluded that the immobile fraction is systematically photobleached very strongly and thus tends to be underestimated. Since Eq 49 is a linear equation, it is invariant under linear scaling while affine scaling is not compatible with this kinetic model. The scaled FRAP equation for binding diffusion kinetics by Wu et al. is summarized in Table 2.

Eq 49 is approximation of a partitioning type binding diffusion equation, when cytosolic diffusion is much faster than binding kinetics so that *u* can reach equilibrium almost immediately before the binding kinetics occurs [31], where a partitioning type binding diffusion equation is described as

$$\begin{array}{c} \left\{ \begin{array}{cc} u_{t}= & D_{u}\Delta u-k_{on}u+k_{off}c\\ b_{t}= & D_{b}\Delta b+k_{on}u-k_{off}c \end{array}\right. \end{array}$$ (50)

Similar to Wu’s model, binding diffusion equations are not also fully compatible with affine and linear scaling. To see this, consider a linear operator for the partitioning type binding diffusion equation (Eq 50),

$$\begin{array}{c} {𝓛}_{BD}=\frac{\partial }{\partial t}-[\begin{array}{cc} D_{u} & 0\\ 0 & D_{b} \end{array}]\Delta -[\begin{array}{cc} -k_{on} & k_{off}\\ k_{on} & -k_{off} \end{array}]. \end{array}$$ (51)

If *u* and *b* represent the soluble and membrane bound protein concentrations that satisfy the partitioning type binding equation, then for constants *c*_1_ and *c*_2_an affine scaling $c_{1}\left(\begin{array}{c} u\\ b \end{array}\right)+c_{2}$ satisfies

$$\begin{array}{ccc} {𝓛}_{BD}(\begin{array}{c} c_{1}u+c_{2}\\ c_{1}b+c_{2} \end{array}) & = & c_{1}{𝓛}_{BD}(\begin{array}{c} u\\ b \end{array})+{𝓛}_{BD}(\begin{array}{c} c_{1}\\ c_{2} \end{array})\\ & = & \left(\begin{array}{c} k_{on}c_{1}-k_{off}c_{2}\\ -k_{on}c_{1}+k_{off}c_{2} \end{array}\right) \end{array}$$ (52)

which doesn’t vanish unless *c*_2_/*c*_1_ = *k*_*on*_/*k*_*off*_. This indicates that affine scalings of *u* and *b* do not satisfy the binding diffusion equation and therefore a partitioning type binding equation is not invariant under affine scaling. In contrast, a linear scaling (*u*, *b*) ↦ (*cu*, *cb*) for the scale scaling factor *c* for both *u* and *b* is compatible with the binding diffusion equation.

On the other hand, many protein kinetics are described by ligand-receptor type reaction diffusion equation as

$$\begin{array}{c} \left\{ \begin{array}{cc} u_{t}= & D_{u}\Delta u-k_{on}u\cdot r+k_{off}c\\ r_{t}= & D_{r}\Delta r-k_{on}u\cdot r-k_{off}c\\ c_{t}= & D_{c}\Delta r+k_{on}u\cdot r-k_{off}c \end{array}\right. \end{array}$$ (53)

where *u*, *r*, and *c* are ligand, receptor, and ligand-receptor complex concentrations. However, Eq 53 is a non-linear equation and therefore is incompatible with either linear, affine, or exponential scaling. Moreover, a closed form of the FRAP equation for Eq 53 is not available to our knowledge. Although we only considered diffusion FRAP on the plane assuming cell membrane geometry can be approximated by infinite plane (ℝ^2^), some FRAP equations have been developed by considering a specific geometry where protein kinetics occur [33–35]. However, a valid scaling is defined as when the scaled variable and the original variable satisfy the same kinetics equation. Thus, the same scaling rules hold regardless of the geometry the FRAP equations are based on, although boundary conditions should be adjusted according to the scaling rules.

One major assumption behind the infinite plane (ℝ^2^) approximation of the plasma membrane is that fluorescence loss during the photobleaching is minimal compared to the total fluorescence. The validity of this assumption can be easily checked by comparing the fluorescence right before and right after the intentional photobleaching event in the whole image fluorescence, *F*_*Whole*_(*t*). If fluorescence loss during photobleaching is minimal, the level of fluorescence observed in the whole image fluorescence will be similar right before and after photobleaching, as illustrated in Fig 1B. While fluorescence loss is very small in the experimental FRAP protocol used in the current study, experiments using larger bleaching regions might suffer from a problem in which full recovery cannot be observed for large bleaching regions and the fluorescence loss due to the intentional photobleaching might be mistaken for an immobile fraction. Such behavior is evidenced by a significant drop in whole image fluorescence immediately after the photobleach. In this case, the loss of fluorescent material due to photobleaching can be corrected by normalizing the data by the average fluorescence intensity of the whole image before and after the bleach. Thus, normalizing the FRAP data by the whole image fluorescence has another beneficial effect in addition to correcting for photofading: it corrects the FRAP curve for the loss of fluorescence caused by the intentional photobleaching step.

Another important assumption in this study is that anomalous diffusion is homogeneous in space and is unrelated to the cellular structure or underlying volume. However, inhomogeneity, for example due to more complex morphologies of the cell membrane, is an important factor that can cause deviations from anomalous diffusion model due to time dependent diffusion coefficient, *D*(*t*)(Eq 3).

In summary, care must be exercised when scaling and normalization schemes are applied in FRAP analysis to correct for photofading and/or a mobile fraction, because applying incompatible scaling or an incorrect sequence of scaling may cause erroneous results. Based on our analysis, we propose a workflow for FRAP data analysis using free diffusion or anomalous diffusion models as described in Fig 7.
